# Risk of SARS-CoV-2 infection in professional settings, shops, shared transport, and leisure activities in France, 2020–2022

**DOI:** 10.1186/s12889-024-19651-y

**Published:** 2024-09-04

**Authors:** Simon Galmiche, Tiffany Charmet, Arthur Rakover, Olivia Chény, Faïza Omar, Christophe David, Alexandra Mailles, Fabrice Carrat, Arnaud Fontanet

**Affiliations:** 1 Emerging Diseases Epidemiology Unit, Institut Pasteur, Université Paris Cité, 25 rue du Docteur Roux, Paris, 75015 France; 2 Sorbonne Université, Ecole Doctorale Pierre Louis de Santé Publique, Paris, 75006 France; 3 Clinical Research Coordination Office, Institut Pasteur, Université Paris Cité, Paris, 75015 France; 4 Department of Public Affairs – Public Statistics, Institut Ipsos, Paris, 75013 France; 5 Santé Publique France, Saint-Maurice, 94410 France; 6 Sorbonne Université, Inserm, IPLESP, Hôpital Saint-Antoine, AP-HP, Paris, 75012 France; 7 Unité PACRI, Conservatoire National des Arts et Métiers, Paris, 75003 France

**Keywords:** SARS-CoV-2, Case-control studies, Infectious disease transmission, Occupational exposure, Workplace, Travel, Leisure activities

## Abstract

**Purpose:**

The aim of the study was to identify settings associated with SARS-CoV-2 transmission throughout the COVID-19 pandemic in France.

**Methods:**

Cases with recent SARS-CoV-2 infection were matched with controls (4:1 ratio) on age, sex, region, population size, and calendar week. Odds ratios for SARS-CoV-2 infection were estimated for nine periods in models adjusting for socio-demographic characteristics, health status, COVID-19 vaccine, and past infection.

**Results:**

Between October 27, 2020 and October 2, 2022, 175,688 cases were matched with 43,922 controls. An increased risk of infection was documented throughout the study for open-space offices compared to offices without open space (OR range across the nine periods: 1.12 to 1.57) and long-distance trains (1.25 to 1.88), and during most of the study for convenience stores (OR range in the periods with increased risk: 1.15 to 1.44), take-away delivery (1.07 to 1.28), car-pooling with relatives (1.09 to 1.68), taxis (1.08 to 1.89), airplanes (1.20 to 1.78), concerts (1.31 to 2.09) and night-clubs (1.45 to 2.95). No increase in transmission was associated with short-distance shared transport, car-pooling booked over platforms, markets, supermarkets and malls, hairdressers, museums, movie theatres, outdoor sports, and swimming pools. The increased risk of infection in bars and restaurants was no longer present in restaurants after reopening in June 2021. It persisted in bars only among those aged under 40 years.

**Conclusion:**

Closed settings in which people are less likely to wear masks were most affected by SARS-CoV-2 transmission and should be the focus of air quality improvement.

**ClinicalTrials.gov (03/09/2022):**

NCT04607941.

**Supplementary Information:**

The online version contains supplementary material available at 10.1186/s12889-024-19651-y.

## Introduction

Identifying settings where transmission of SARS-CoV-2 occurs and quantifying their respective contribution has been central to advise evidence-based mitigation strategies such as social distancing policy, testing practices, contact tracing, and information to the public [1]. As the impact of the pandemic recedes in most countries, knowledge on the settings of transmission can help guide improvement in air quality and individual protection approaches, particularly for elderly or immunocompromised people. Furthermore, drawing all available information from the SARS-CoV-2 pandemic is essential to preparedness efforts: in case of emergence of a new respiratory virus, knowledge derived from SARS-CoV-2 will support a timely and evidence-based public health response.

Throughout the pandemic, numerous factors potentially affecting where SARS-CoV-2 transmission may occur have undergone significant changes, including non-pharmaceutical interventions, vaccine coverage, or the circulating strain.

Several study designs have been used to address this question. Outbreak reports have generated crucial early evidence, particularly in settings with low community transmission, allowing accurate contact tracing [2–6]. Other studies have aimed to screen all contacts of a series of cases and identify in which settings contacts were more likely to result in transmission [7, 8]. These designs often require the correct identification of contacts, which can be difficult for SARS-CoV-2 in case of long-distance airborne transmission [9] or superspreading event: a study in Hong Kong reported that approximately 20% of cases were responsible for 80% of secondary cases [10]. It is especially challenging in locations where unrelated people interact closely, for instance in public transport. Other studies have estimated the risk or odds ratios of infection associated with different settings, through cross-sectional seroprevalence estimates [11–13] or case-control designs [14–17].

While many studies have identified the risk associated with venues such as bars, night-clubs, or public transport, none have provided a long-term outlook to assess potential changes through the pandemic. In the present study, which was conducted over a two-year period, we used a case-control design to identify settings associated with the risk of SARS-CoV-2 infection in France and assess how these evolved through the pandemic.

## Methods

We conducted an online case-control study in mainland France from October 2020 to October 2022. The methods of the study have been reported before [18–20]. We included cases aged 18 and above with recently diagnosed SARS-CoV-2 infection reported in a national information system: all cases diagnosed through RT-PCR or rapid antigen tests were centralized by the national health insurance system (*Caisse nationale d’assurance maladie*, CNAM). The CNAM sent email invitations to cases identified within the past week who had previously provided their email address (approximately 55% of all people affiliated with the CNAM, who represent about 89% of the population of mainland France). Both RT-PCR and rapid antigen tests were available free of charge without prescription for the whole duration of the study. After providing consent, participants completed an online questionnaire about sociodemographic information, health status, household description, and recent exposures of interest. The questions focused on the 10 days preceding the onset of the symptoms (or testing if asymptomatic). This period was reduced to 7 days after the emergence of the omicron variant given its shorter incubation period [21]. Following the participation of the cases, controls were enrolled by Ipsos, a market and opinion research company, and matched with cases using a frequency-matched procedure. Matching criteria were age (18–29, 30–54, ≥ 55 years old), sex (male or female as self-reported), region (largest administrative subnational division), size of population in the area of residence, and week of exposure to account for local transmission dynamics.

We did not include potential cases and controls who were under a legal status of curatorship or guardianship at the time of participation. Until February 2021, we included only controls without a past episode of SARS-CoV-2 infection. Eligibility for controls was then extended to people without ongoing SARS-CoV-2 infection. We excluded cases and controls reporting an episode of SARS-CoV-2 infection in the past two months (other than the one leading to their participation for cases). To limit recall errors, we excluded cases who filled the questionnaire over 30 days after the onset of symptoms (or testing if asymptomatic). We allowed repeated participation after at least two months since the last participation from January 2022.

To study the evolution of the risk associated with the exposures of interest over the course of the study, we divided the study period into nine shorter periods, based on incidence, important non-pharmaceutical interventions (stay-at-home orders, curfews, sanitary pass, i.e. a proof of COVID-19 vaccination, past infection, or a recent negative test required to visit a series of places), and the circulating strain (see supplementary methods for further description of periods and non-pharmaceutical interventions throughout the study).

### Statistical analysis

For a better matching of controls with cases on the timing of exposure, considering that controls were initially included after the screening of cases, we proceeded to an exact matching procedure of four cases for one control on the calendar week of the outcome date (symptom onset or testing if asymptomatic for cases, questionnaire completion for controls). To account for the random selection of cases in the matching procedure, as those outnumbered controls more than four times, we generated 100 databases of series of four cases matched with one control for each period.

We fitted unconditional logistic regression models to estimate the odds ratios of SARS-CoV-2 infection for the exposures of interest in a model including the matching variables, as well as health-related variables, including COVID-19 vaccine status, past SARS-CoV-2 infection, sociodemographic characteristics (level of education, socio-professional category) and household description. The choice of the adjusting variables was guided by subject matter knowledge to include all measured causes of the exposures, the outcome (SARS-CoV-2 infection), or both, relying on the disjunctive cause criterion [22]. The exposures of interest were included as follows: workplace (work in an office, open-space arrangement, complete or partial remote office), gatherings (professional, private, or religious), retail settings (shops, hairdresser, beauty salon), shared transport (short-distance or long-distance transport, car-pooling), and leisure activities (cultural venues, sports facilities, bars, restaurants, parties, divided into night-clubs or private parties from period 5 onward). No estimate was produced for bars, restaurants, indoor sports facilities, and cultural venues for periods 2 and 3 as they were mostly closed then (odds ratios were estimated for the first period thanks to the inclusion of participants before the start of the stay-at-home orders).

These models were fitted for each of the 100 databases per period. We extracted the coefficients, exponentiated them and retained the median for the point estimate and the 2.5th and 97.5th percentiles for the 95% confidence interval. In complementary models, we explored potential interactions of bars, restaurants, and parties with age categorized as < 40 or ≥ 40 years (*p*-value for interaction estimated with the median of the 100 estimates). To investigate how viral circulation in the country of destination could affect the risk associated with airplane travel, we calculated the mean daily incidence rate obtained from Ourworldindata.org in the country of destination over period 4 (summer of 2021, emergence of the delta variant) [23].

All statistical analyses were performed using Stata 16.0 (StataCorp, College Station, USA). (See supplementary methods for further description of statistical analysis.)

### Ethics approval

This study received ethics approval from the ethics committee *Comité de Protection des Personnes Sud Ouest et Outre Mer 1* on September 21, 2020 as required by French regulation on clinical research. The data protection authority, the *Commission Nationale de l’Informatique et des Libertés* (CNIL) authorized the processing of data on October 21, 2020. Informed consent was obtained from all participants.

This report follows the STROBE reporting guidelines for observational studies.

## Results

**Fig. 1:**
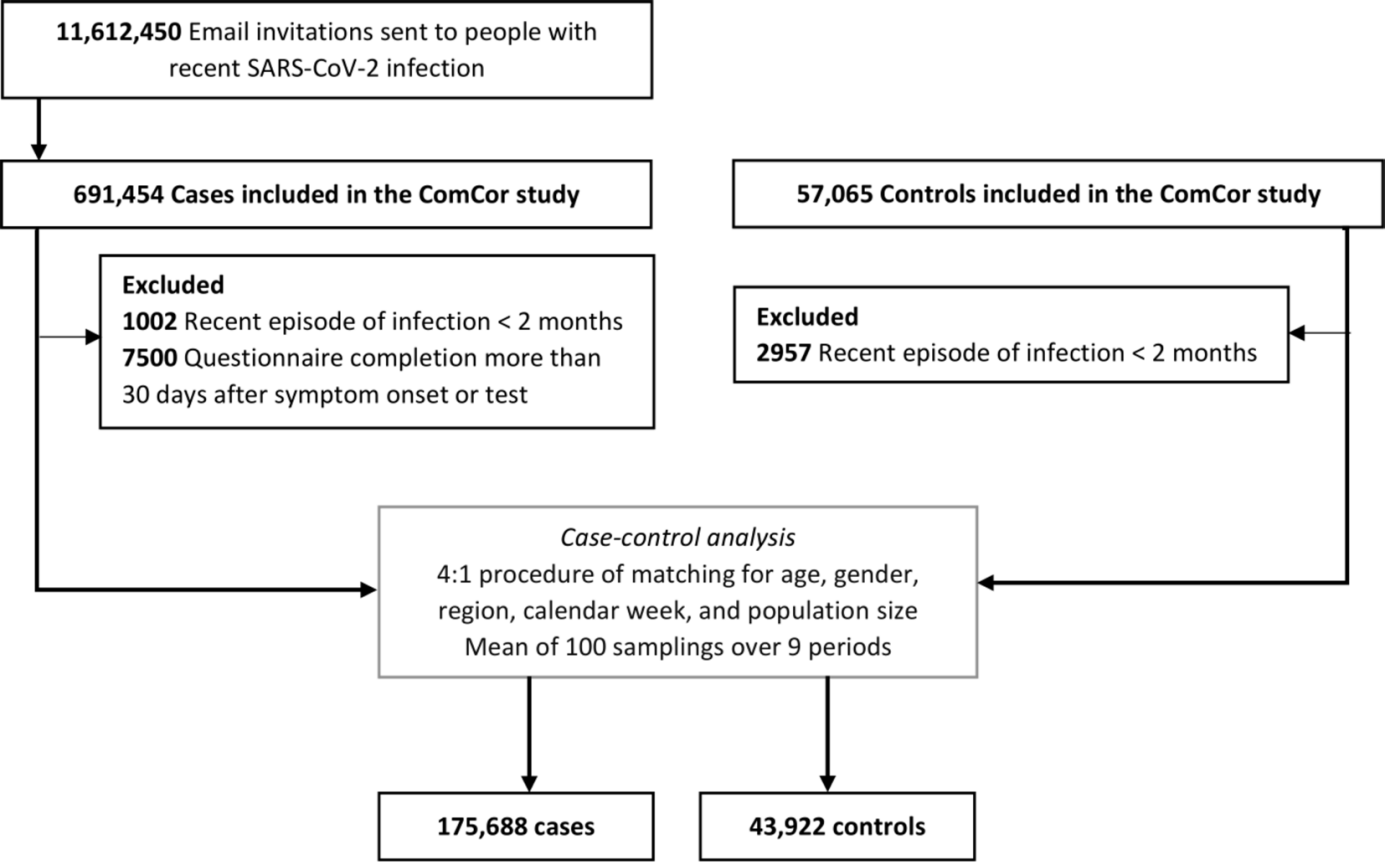
Flow chart of participant enrollment and matching of cases and controls. Legend: Study conducted in mainland France between October 2020 and October 2022

From October 27, 2020 to October 2, 2022, we sent 11,612,450 email invitations to people with recent SARS-CoV-2 infection, and included 691,454 cases (6.0%) and 57,065 controls. After exclusion of participants with a reported episode of infection in the past two months and cases who responded to the questionnaire over 30 days after symptom onset, and matching of four cases for one control, we included 175,688 cases and 43,922 controls (Fig. 1). The main socio-demographic and health status characteristics are summarized in Table 1. The study population was characterized by a lower proportion of men (33.8% vs. 47.6% in the general population aged 20 and over in France), a higher representation of people aged between 40 and 49 years (26.8% vs. 16.7%) and of residents of the Ile-de-France region (where Paris is located) (22.7% vs. 18.3%).

**Table 1:** Socio-demographic description of the study population (case-control study in mainland France, October 2020 to October 2022)

Total	Cases, *n* (%)	Controls, *n* (%)	*p*-value
	175,688	43,922	
**Male sex**	59,380 (33.8)	14,845 (33.8)	> 0.99
**Age (years)**			
18–29	21,244 (12.1)	5311 (12.1)	> 0.99
30–39	31,888 (18.2)	7972 (18.2)	
40–49	47,032 (26.8)	11,758 (26.8)	
50–59	37,960 (21.6)	9490 (21.6)	
60–69	24,796 (14.1)	6199 (14.1)	
≥ 70	12,768 (7.3)	3192 (7.3)	
**Population in the area of residence**			
< 5000 inhabitants	44,828 (25.5)	11,207 (25.5)	> 0.99
5000–19,999 inhabitants	13,984 (8.0)	3496 (8.0)	
20,000–99,999 inhabitants	17,800 (10.1)	4450 (10.1)	
Over 100,000 inhabitants	61,956 (35.3)	15,489 (35.3)	
Greater Paris area	37,120 (21.1)	9280 (21.1)	
**Region of residence**			
Ile-de-France	39,848 (22.7)	9962 (22.7)	> 0.99
Auverge-Rhône-Alpes	23,384 (13.3)	5846 (13.3)	
Occitanie	17,860 (10.2)	4465 (10.2)	
Provence-Alpes-Côte d’Azur and Corsica	15,788 (9.0)	3947 (9.0)	
Grand Est	15,336 (8.7)	3834 (8.7)	
Nouvelle-Aquitaine	15,132 (8.6)	3783 (8.6)	
Hauts-de-France	14,800 (8.4)	3700 (8.4)	
Pays de la Loire	8580 (4.9)	2145 (4.9)	
Bretagne	8324 (4.7)	2081 (4.7)	
Normandie	6124 (3.5)	1531 (3.5)	
Bourgogne-Franche-Comté	5876 (3.3)	1469 (3.3)	
Centre-Val de Loire	4636 (2.6)	1159 (2.6)	
**Education level**			
No diploma	3682 (2.1)	767 (1.7)	< 0.001
Pre-high school diploma	25,995 (14.8)	7722 (17.6)	
High-school diploma	31,741 (18.1)	10,559 (24.0)	
Bachelor’s degree	62,909 (35.8)	16,340 (37.2)	
Master’s degree or higher	45,149 (25.7)	7056 (16.1)	
Missing	6212 (3.5)	1478 (3.4)	
**Health conditions**			
Diabetes mellitus	6157 (3.5)	2304 (5.2)	< 0.001
Hypertension	20,476 (11.7)	5773 (13.1)	< 0.001
Chronic respiratory disease	14,650 (8.3)	3132 (7.1)	< 0.001
Coronary artery disease	2114 (1.2)	431 (1.0)	< 0.001
**Body-mass index (kg/m²)**			
Healthy weight (≥ 18.5 & <25)	89,392 (50.9)	20,909 (47.6)	< 0.001
Underweight (< 18.5)	5409 (3.1)	1880 (4.3)	
Overweight (≥ 25 & <30)	52,824 (30.1)	13,111 (29.9)	
Obesity (≥ 30)	28,061 (16.0)	8023 (18.3)	
**Housing**			
Individual house	106,786 (60.8)	25,775 (58.7)	< 0.001
Apartment	68,223 (38.8)	18,004 (41)	
Shelter	585 (0.3)	118 (0.3)	
Nursing home	92 (0.1)	25 (0.1)	
**Living with a child**			
Attending daycare	4946 (2.8)	928 (2.1)	< 0.001
Attended for by a professional in-home caregiver	5772 (3.3)	904 (2.1)	< 0.001
Attending preschool	16,467 (9.4)	3327 (7.6)	< 0.001
Attending primary school	29,027 (16.5)	6185 (14.1)	< 0.001
Attending middle school	26,795 (15.3)	6183 (14.1)	< 0.001
Attending high school	21,990 (12.5)	5447 (12.4)	0.54
Attending university	14,711 (8.4)	4075 (9.3)	< 0.001
**Past SARS-CoV-2 infection**			
No past infection	165,798 (94.4)	37,376 (85.1)	< 0.001
Past infection 61–180 days	8085 (4.8)	2968 (7.1)	
Past infection > 180 days	1805 (1.1)	3578 (8.5)	
**COVID-19 vaccine**,** time since last dose**			
Unvaccinated	49,989 (28.5)	13,503 (30.7)	< 0.001
1 dose, < 90 days	3288 (2.0)	870 (2.1)	
1 dose, 90–179 days	552 (0.4)	260 (0.7)	
1 dose, > 179 days	615 (0.4)	264 (0.8)	
2 doses, < 90 days	7301 (4.9)	3024 (8.1)	
2 doses, 90–179 days	13,718 (9.2)	3150 (8.5)	
2 doses, > 179 days	7254 (5.2)	2092 (6.0)	
3 doses, < 90 days	26,324 (17.7)	6794 (18.3)	
3 doses, 90–179 days	40,047 (32.4)	6810 (22.0)	
3 doses, > 179 days	16,697 (13.5)	2925 (9.5)	
4 doses, < 90 days	2728 (2.4)	560 (2.0)	
Missing date of last dose	7162 (4.8)	3669 (9.9)	
**Study period (onset date)**			
1 (10/01/2020)	7308 (4.2)	1827 (4.2)	
2 (12/04/2020)	19,636 (11.2)	4909 (11.2)	
3 (04/09/2021)	9008 (5.1)	2252 (5.1)	
4 (06/14/2021)	11,264 (6.4)	2816 (6.4)	
5 (08/14/2021)	4820 (2.7)	1205 (2.7)	
6 (10/02/2021)	11,248 (6.4)	2812 (6.4)	
7 (12/20/2021)	44,136 (25.1)	11,034 (25.1)	
8 (03/18/2022)	39,652 (22.6)	9913 (22.6)	
9 (05/20/2022) (end date: 10/02/2022)	28,616 (16.3)	7154 (16.3)	

**Fig. 2:**
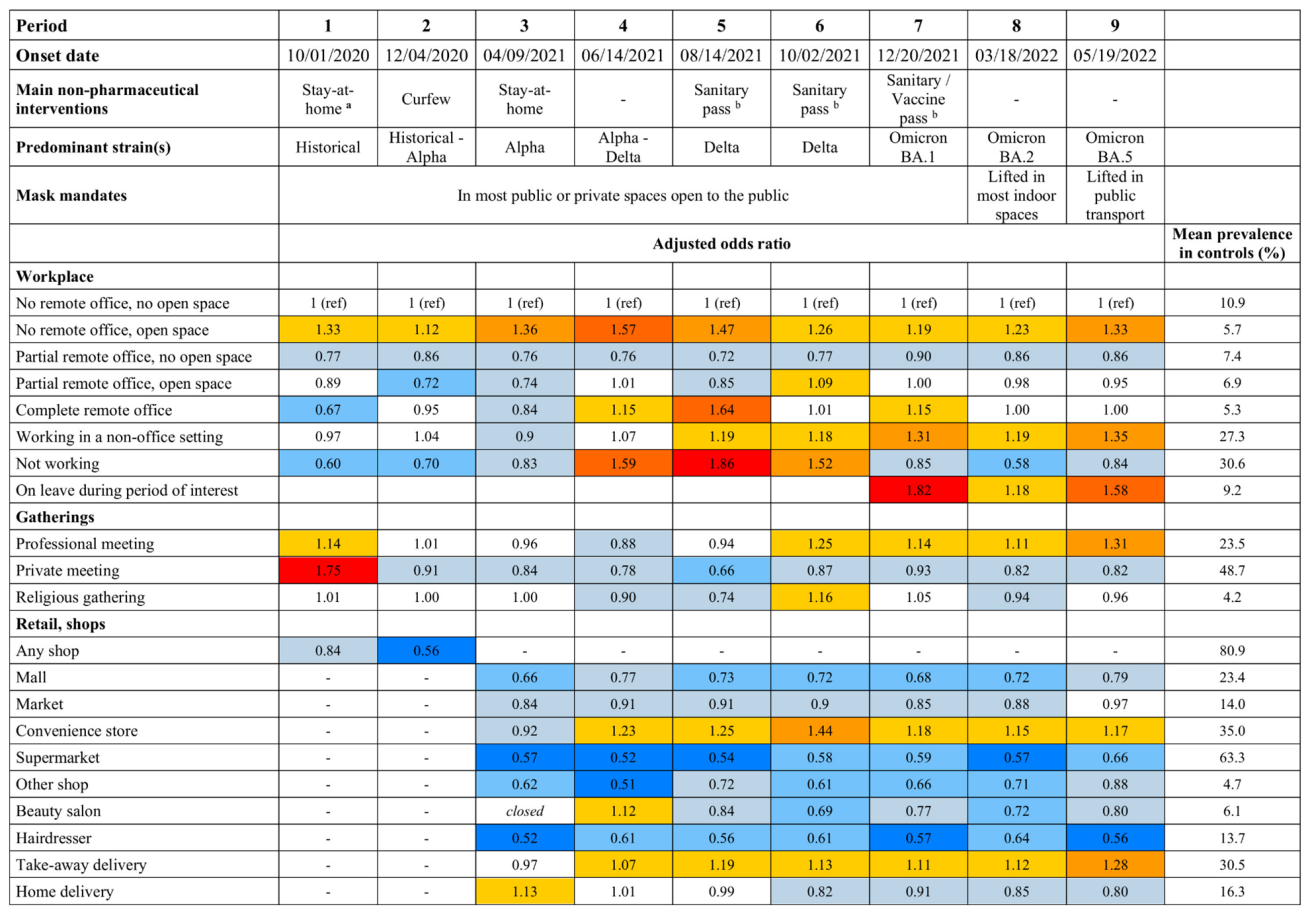

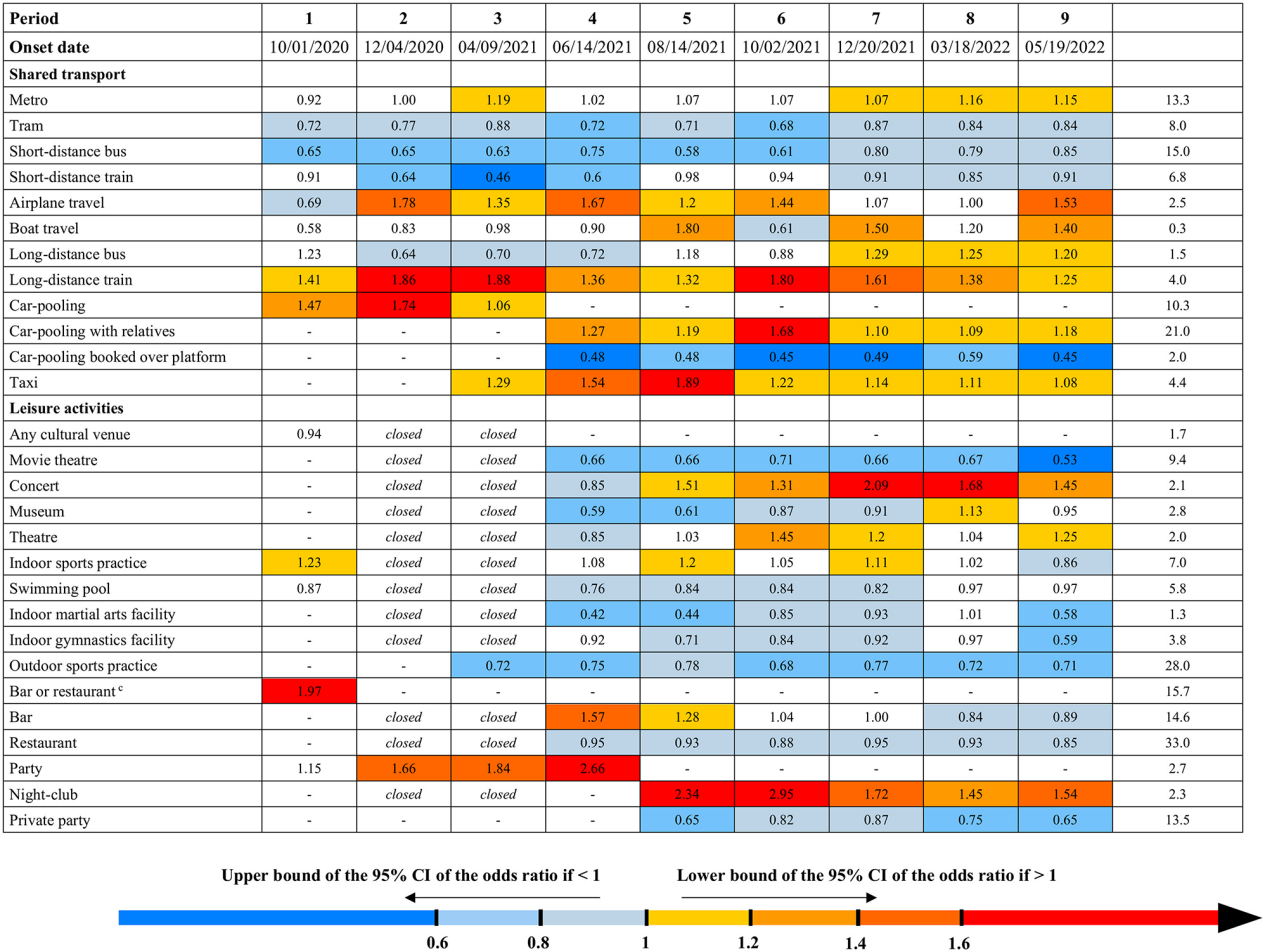
Adjusted odds ratios of SARS-CoV-2 infection in a case-control study in France. Legend: The study period was divided in nine shorter study periods based on incidence, the circulating strains (two strains are indicated when the period includes the rise of a new strain), and the main non-pharmaceutical interventions. The colors of the cells refer to 95% confidence intervals’ width: in shades of blue if the upper bound of the 95% CI is < 1, in shades of red if the lower bound of the 95% CI is > 1. Cells are uncolored if the 95% confidence interval includes 1. The empty cells reflect the modifications of the questionnaire through the course of the study. Cases and controls were matched with a 4:1 ratio on sex (female or male), age (in 10-year-age categories), region, size of population of the area of residence, and week of exposure. To account for random selection of cases as those outnumbered controls more than four times, we generated 100 databases for each period with matched sets of 4 cases per control. The odds ratios were estimated in multivariable logistic regression models for each of the periods, adjusting for all the variables shown in the figure as well as the matching variables, household characteristics (number of people in the household, presence of children, type of housing), professional category, health status (body-mass index, smoking status, hypertension, diabetes mellitus, chronic respiratory disease, coronary artery disease, immunosuppression), past episode of infection (categorized as 61–180 days prior or > 180 days prior), and COVID-19 vaccine status (number of doses and time since last dose divided in < 90 days, 90–179 days, ≥ 180 days). The odds ratio and the 95% confidence intervals estimates were inferred through the 50th, 2.5th, and 97.5th percentiles of the 100 estimates for each period. All variables shown here are dummy variables except for one combined variable regarding the workplace. (**a**): The stay-at-home orders started on 10/30/2020; bars, restaurants, night-clubs, non-essential shops, and cultural venues were then closed. (**b**): The sanitary pass was a proof of vaccine, past infection, or a recent negative test to enter a series of public spaces; the vaccine pass was implemented on 01/24/2022 to include only proofs of vaccine or past infection. (**c**): Estimated in people exposed before the start of the stay-at-home orders on 10/30/2020

We identified several settings associated with an increased risk of infection, including professional settings, shops, shared transport, and leisure activities (Fig. 2, Tables S1-S3).

Regarding the workplace, we found a consistently increased risk associated with working in an open-space office compared with a non-open-space office environment (OR range through the nine periods of the study: 1.12 to 1.57). Remote office was associated with a decreased risk of infection when done only partially in the preceding days (0.72 to 0.90), but often not when the few days spent at the workplace were in an open-space environment (0.72 to 1.09). The risk varied through the study for people reporting working fully remotely, with an OR ranging between 0.67 and 1.64 depending on the period.

The visit of shops was overall not associated with any increased risk of infection. The only exceptions are convenience stores and take-away deliveries for which the risk remained increased through most of the study (periods 4 to 9, OR range: 1.15 to 1.44, and 1.07 to 1.28, respectively). Notably, we found no increased risk in retail facilities involving closer and longer contacts such as hairdressers or beauty salons.

Analyses on shared transport show that most short-distance transport such as buses, tramways or short-distance trains did not increase the risk of infection, except for the metro in which the risk was regularly moderately increased (periods 3, and 7 to 9, OR range: 1.07 to 1.19). On the other hand, long-distance trains and airplanes were associated with a notably increased risk, consistently for train (1.25 to 1.88) and for most of the study for airplanes (periods 2 to 6, and 9, OR range: 1.20 to 1.78). The models were adjusted on abroad travel, suggesting that the effect for airplane was not mediated through visit to a high-incidence country. In a sensitivity analysis, the risk for airplane travel remained increased after adjustment on the mean incidence in the country of destination (period 4: OR 1.58, 95%CI 1.41–1.78, compared with OR 1.67, 95%CI 1.52–1.88 without adjustment). Car travels also appeared to favor transmission in certain circumstances, with increased risks for taxi rides throughout the study in the periods when they were investigated (periods 3 to 9, OR range: 1.08 to 1.89), as well as for car-pooling, but only when traveling with relatives (periods 4 to 9, OR range: 1.09 to 1.68), not when the car-pooling was organized with unrelated persons through a dedicated platform (periods 4 to 9, OR range: 0.45 to 0.59).

Of all the cultural and sports facilities we investigated, we found an increased risk mainly for the attendance of concerts (periods 5 to 9, OR range: 1.31 to 2.09), and less consistently for theatres (periods 6, 7, and 9, OR range: 1.20 to 1.45) and the practice of sports indoors (periods 1, 5, and 7, OR range: 1.11 to 1.23). We found no increased risk associated with other settings such as museums, movie theatres, swimming pools, or martial arts facilities.

We initially identified an increased risk associated with bars and restaurants (as the questionnaire did not distinguish them at first): period 1, OR 1.97 (95%CI 1.84–2.07). As bars and restaurants reopened in the spring of 2021, we found an increased risk for bars (OR 1.57, 95%CI 1.50–1.64) but not for restaurants (OR 0.95, 95%CI 0.89–0.99). The risk gradually decreased afterward for bars, and they were no longer at risk from October 2021 (period 6) onward. However, there were significant interactions of bars with age categorized as under 40 years or 40 and above, with a persistently increased risk in those aged under 40 until the first omicron BA.1 wave (periods 4 to 7, OR range: 1.23 to 2.17) (Tables S5, S6). Attending parties was initially consistently associated with an increased risk, particularly in those aged under 40 (periods 1 to 4, OR range: 1.33 to 3.24) (Tables S4, S5). When we could distinguish private parties from parties in night-clubs, as those reopened in the summer of 2021, we found no increased risk for private parties (no interaction with age, Tables S5, S6). In contrast, the risk was high for night-clubs (e.g., in period 6, OR 2.95, 95%CI 2.64–3.28). It decreased gradually through the various omicron waves in 2022 but remained increased in the last period of the study (omicron BA.4/5 wave, OR 1.54, 95%CI 1.41–1.66), regardless of the age category (Tables S5, S6).

## Discussion

This case-control study provides a long-term perspective on the settings most associated with the risk of SARS-CoV-2 infection in mainland France between October 2020 and October 2022. We identified increased risks for on-site office work, particularly in open space environments, professional meetings, concerts, theatres, long-distance public transit, as well as bars and night-clubs. On the other hand, we found no increased risk for most sports and cultural activities, religious gatherings, shops, and short-distance public transport.

All settings associated with an increased risk of infection in our study are characterized by varying degrees of common characteristics: mostly indoor settings with little air renewal, where contacts are close, numerous, often maskless, sometimes including singing or shouting, and usually last more than a mere few minutes. These factors are in line with knowledge on the conditions allowing transmission of SARS-CoV-2, through direct contact, large droplets, or fine aerosols [9, 24], often indoors [25].

Apart from bars, restaurants, and night-clubs for which the risk decreased through the course of the study, the associations remained overall relatively stable. Following the implementation of the sanitary pass in most indoor places and long-distance transport in August 2021 (period 5 onwards), we observed a slight decrease in the risk associated with airplane travel, long-distance train, and bars, suggesting the sanitary pass contributed to decrease the risk of transmission in those environments. The odds ratios increased again in period 6 (October 2 to December 19, 2021). While this increase must be interpreted with caution given its limited amplitude, it may also result from a rapidly waning vaccine effectiveness on SARS-CoV-2 transmission [26], as the majority of the adult population was vaccinated between June and August 2021. Other factors could explain the decrease of the risk for bars and restaurants: night-clubs were closed from March 2020 until July 2021, and people may have been more prone to attend parties with closer and longer interactions in bars and restaurants during that time than in the later periods. The interaction of parties and bars with age, with higher odds ratios observed for people aged under 40, suggests the role of behavioral patterns in those environments. The summer of 2021 was also characterized by an important football European competition, during which public viewing in bars was popular and likely contributed to viral circulation. Another hypothesis is that the transmission of the more contagious delta and omicron variants did not require as favorable conditions as the previous strains, leading to a less differentiated risk between people visiting these settings and people who avoided them. Studies on the evolution of settings of transmission through the emergence of the various strains in other countries would help explore this hypothesis.

Findings of other studies on settings of transmission are overall consistent with ours. The decreased risk for people reporting working remotely was also shown in several other studies [16, 27–29], whereas the role of open-space offices has been little studied [30]. The fact that people working in open-space offices while also working partially remotely were not at lower risk of infection suggests that the benefit of remote office was offset by the increased risk in open-space offices. The varying results for complete remote office suggest residual confounding in our analysis; these people were possibly exposed to SARS-CoV-2 transmission in other settings that they were more likely to visit, or in the household, in ways that our models could not account for. The absence of increased risk for retail facilities, as well as hairdressers and beauty salons, also reported by others [14–17, 27, 31–33], suggests that these facilities had low enough density and stringent enough measures to effectively limit transmission. Findings on shared transport, often studied altogether without distinction of the duration of the trip, have yielded conflicting evidence [12, 14, 27, 32, 34, 35]. A contact-tracing study on air travel in Ireland has underlined the role of the duration of the flight, with secondary attack rates reaching 14.9% for flights lasting over 5 h vs. 6.3% for shorter ones [36]. The contrast we found between short- and long-distance shared transport supports the importance of the duration spent onboard. Long-distance bus travels were inconsistently at increased risk, which might result from better air renewal during the mandatory driving breaks compared to other shared transport.

Consistent with several other studies, we found no increased risk for cultural venues, sports facilities, and religious gatherings, apart from a slightly increased risk in theaters, concerts, and indoor sports facilities [14, 15, 17, 27, 31, 37]. Findings on dining and partying venues often reported an increased risk for bars [14, 15, 17, 27, 38, 39], night-clubs and parties [17, 27, 39], whereas findings are more conflicted for restaurants [15, 27, 31, 32, 38, 40]. In a case-control study in Denmark conducted in June 2021, Munch and colleagues found an increased risk for the attendance of restaurants or cafés only for people reporting alcohol consumption [31]. This highlights the role of behaviors within those settings on the risk of transmission. Two randomized trials conducted in France found no increased risk of SARS-CoV-2 infection in participants of mass gatherings with strict requirements for attendance, one at a concert [41] and one in a nightclub [42]. These experimentations offer clues for continuation of mass indoor gatherings in case of an emerging respiratory pathogen.

Our findings suggest a graduation in the risk of transmission which combines degree of air renewal and behaviors. We did not observe excess transmission in indoor places where consistent mask-wearing was possible such as museums, movie theatres, shopping malls, or beauty salons. In bars and restaurants, reopening in mid-2021 was associated with recommendations for spacing tables and opening doors and windows. In this context, increased risk of transmission was observed only for those younger than 40 years of age, or during special events like the European soccer championship, suggesting that opening doors and windows might have been protective provided individuals avoided staying too close to one another, and talking loudly or shouting. In closed spaces where opening doors and windows may be absent or limited, and mask wearing not maintained systematically (e.g., during meals in long-distance trains, or drinks in night-clubs), increased risk of transmission was observed. In such places, investment in improving air renewal or filtration would be essential to improve air quality and decrease transmission risk. These investments are costly but have potential long-term benefits on population health [43] and should be properly evaluated for their feasibility and effectiveness.

The main limitation of the present study lies in the low participation rate (6.0%). As in other studies conducted online, we observed notable differences between our study population and the source population: we included a more female population, often in intermediary age groups, and with a higher education level [44, 45]. These demographic factors were included in our matching procedure or in the adjusted models, thus limiting the risk of recruitment bias. This does however decrease the generalizability of our findings. Furthermore, unexpected findings such as the intermittently increased risk associated with complete remote office, with take-away delivery, or the decreased risk for supermarkets illustrate potential recruitment bias, residual confounding, or result from the multiplicity of comparisons. These biases are difficult to avoid entirely in a case-control study. We chose this design nonetheless as it enabled us to modify the questionnaire whenever necessary (introducing questions on the vaccine status, variants, etc.). It provided results soon after the beginning of the study which helped evidence-based decision-making in a time of high burden of SARS-CoV-2 in France [18, 20]. Since the incubation period of COVID-19 only lasts a few days, we considered the risk of recall bias, a frequent limitation of case-control studies, would be low, although we cannot rule out that cases recalled exposures more precisely than controls as they retrospectively tried to identify the circumstances of infection. Cases and controls differed significantly regarding past infection status, an inclusion bias whose impact was likely mitigated by the adjustment on past infection status in the multivariable models. The consistency of the present findings with those of other studies using different designs, such as cross-sectional seroprevalence estimates or prospective cohorts, also supports the validity of our approach. Despite significant power, our study could not assess finer exposures or behaviors associated with certain settings. We cannot exclude for instance that the increased risk associated with train or air travel was caused by exposures at the train station or the airport, or that specific partying venues might have been safe provided they implemented distancing or testing practices.

Overall, this case-control study shows that the workplace, long-distance shared transport, and several leisure activities were likely settings of effective SARS-CoV-2 transmission during the pandemic in mainland France. These findings will help focusing efforts on improvement in air quality and inform pandemic preparedness strategies.

## Electronic supplementary material

Below is the link to the electronic supplementary material.

Supplementary Material 1

## Data Availability

The data that support the findings of this study are available from Institut Pasteur. Restrictions apply to the availability of these data, which were used under authorized agreement for this study from the data protection authority, the Commission Nationale de l’Informatique et des Libertés (CNIL). Access to these pseudonymized data would therefore require prior authorization by the CNIL.
