# Supplementary material for: Risk of SARS-CoV-2 infection in professional settings, shops, shared transport, and leisure activities in France, 2020–2022
Source: BMC Public Health. 2024 Sep 4;24:2411. doi: 10.1186/s12889-024-19651-y (PMC11376041; doi:10.1186/s12889-024-19651-y)
Supplement: Supplementary file 1 — Supplementary Material 1 [file 12889_2024_19651_MOESM1_ESM.docx]

**Supplementary information**

**Risk of SARS-CoV-2 infection in professional settings, shops, shared transport, and leisure activities in France, 2020-2022**

Simon Galmiche^1,2^, Tiffany Charmet^1^, Arthur Rakover^1^, Olivia Chény^3^, Faïza Omar^4^, Christophe David^4^, Alexandra Mailles^5^, Fabrice Carrat^6^, Arnaud Fontanet^1,7^

1: Emerging Diseases Epidemiology Unit, Institut Pasteur, Université Paris Cité, 75015 Paris, France

2: Sorbonne Université, Ecole Doctorale Pierre Louis de Santé Publique, 75006 Paris, France

3: Clinical research coordination office, Institut Pasteur, Université Paris Cité, 75015 Paris, France

4: Department of Public Affairs – Public Statistics, Institut Ipsos, 75013 Paris, France

5: Santé Publique France, 94410 Saint-Maurice, France

6: Sorbonne Université, Inserm, IPLESP, Hôpital Saint-Antoine, AP-HP, 75012 Paris, France

7: Unité PACRI, Conservatoire National des Arts et Métiers, 75003 Paris, France

**Corresponding author:** Simon Galmiche, Emerging Diseases Epidemiology Unit, Institut Pasteur, 25 rue du Docteur Roux, 75015 Paris, France

**Phone:** +33140613763

**Email:** simon.galmiche@pasteur.fr

**Supplementary methods**

**Details on important non-pharmaceutical interventions during the study period in France**

The sanitary pass was enforced from August 9, 2021, to March 14, 2022: a proof of COVID-19 vaccination or past infection, or a recent negative SARS-CoV-2 test was required to visit a series of places (long-distance shared transport, restaurants, bars, cultural venues, night-clubs, etc.). A recent negative test was no longer accepted between January 24, 2022 and March 14, 2022 (“vaccine pass”). Night-clubs were closed from the beginning of the study to July 9, 2021, and from December 10, 2021 to February 16, 2022. Both RT-PCR and rapid antigen tests were available free of charge without prescription for the whole duration of the study. The only exception was tests performed to benefit from the sanitary pass, which were no longer free of charge from October 15, 2021 onwards.

**Description of the delimitation of the nine shorter periods used to describe changes through the study**

Period 1 began on October 1, 2020, corresponding to the start of the study period and covered the second wave (historical D614G strain) and the second stay-at-home orders (start on October 30, 2020, less stringent than the first stay-at-home orders). Period 2 began on December 4, 2020 and covered the third wave (alpha variant) and curfew restrictions. Period 3 began on April 9, 2021 and covered the receding alpha wave and the third lockdown (less stringent than the previous two). Period 4 began on June 14, 2021 and covered the fourth wave and the emergence of the delta variant. Period 5 began on August 14, 2021 after implementation of the sanitary pass and covered the receding fourth wave (delta variant). Period 6 began on October 2, 2021 and covered the start of the fifth wave (delta variant). Period 7 began on December 20, 2021 and covered the fifth wave, during which the omicron variant (BA.1 subvariant) predominated. Period 8 began on March 18, 2022 (following the lifting of the mask mandate in most indoor spaces) and covered the omicron BA.2 wave. Period 9 began on May 19, 2022 (following the lifting of the mask mandate in public transport) and covered the omicron BA.4/BA.5 wave, extending to the end of the study period on October 2, 2022.

We added a five- or four-day delay (depending on the predominant strain) between important shifts and the start of the new period to account for the incubation period. We did not perform a priori power calculation when choosing the number of periods. We kept the number of periods relatively low to avoid excessive loss of statistical power for specific periods.

**Data for the general population in France**

Data on the demographic structure of the general population in France in 2021 were obtained from the national institute of statistics and economic studies (data for people aged 20 and over used as approximation of the total adult population) [1].

**Further description of statistical analysis**

During the first period of the study, the timing of exposure used for matching was before or during the second stay-at-home orders. For matching, we used finer age categories (18-29, then 10-year-age categories, until ≥70) than the ones used for initial enrollment of controls (18-29, 29-58, ≥59), along with sex, region, and size of population in the area of residence.

The multivariable models included, beyond the exposures of interest, variables on health status (diabetes, hypertension, respiratory disease, coronary artery disease, immunosuppressive treatment, body-mass index categorized as <18.5 kg/m², [18.5-25[, [25-30[, and ≥30 kg/m²), COVID-19 vaccine status (categorized as number of doses and time since last dose as <90 days, 90-179 days, ≥180 days, with a specific category for participants with missing data for the date of the last dose), past SARS-CoV-2 infection (categorized as 61-180 days prior or over 180 days prior), smoking status, level of education (categorized as no diploma, pre-high school diploma, high school diploma, bachelor’s degree, and Master’s degree or higher) (except in period 1 during which the data was not available for most participants), professional category (of the person in the household with the highest income for the first two periods categorized as employee, intermediate profession, independent profession, senior executive, worker, retired, unemployed/inactive, of the participant for the following periods categorized as employee, intermediate profession, worker/farmer/independent profession, senior executive, retired, unemployed/inactive, students), and household description (housing type, number of people in the household, presence of children).

The questionnaire required answer to the previous questions before advancing. Therefore, we had no missing data, except for the date of the last vaccine injection for some participants (analysed in a separate category).

For the sensitivity analysis regarding airplane travel, we retained the mean incidence rate in the country with the highest incidence if multiple countries were visited and introduced the incidence rate (categorized as ≤ 100 cases per million, ]100-200], ]200-300], ]300-400], and > 400 cases per million) in a similar logistic regression model.

**References**

1. Estimation de la population au 1^er^ janvier 2023 | Insee [Internet]. [cited 2023 Jul 12]. Available from: https://www.insee.fr/fr/statistiques/1893198

**Table S1: Exposure prevalence, odds ratios of SARS-CoV-2 infection in univariable and multivariable estimates in a case-control study conducted in mainland France (2020-2022) – Results for the first three periods of the study (October 1, 2020 to June 13, 2021)**

| Period (onset date) | 1: 10/01/2020 | | | | 2: 12/04/2020 | | | | 3: 04/09/2021 | | | |
| --- | --- | --- | --- | --- | --- | --- | --- | --- | --- | --- | --- | --- |
|  | Cases | Controls | OR (95% CI) | Adjusted OR (95% CI) | Cases | Controls | OR (95% CI) | Adjusted OR (95% CI) | Cases | Controls | OR (95% CI) | Adjusted OR (95% CI) |
| Male sex | 2516 (34.4) | 629 (34.4) | 1.00 (1.00-1.00) | 1.06 (1.02-1.09) | 5904 (30.1) | 1476 (30.1) | 1.00 (1.00-1.00) | 1.04 (1.02-1.07) | 2440 (27.1) | 610 (27.1) | 1.00 (1.00-1.00) | 1.12 (1.09-1.15) |
| **Age (years)** |  |  |  |  |  |  |  |  |  |  |  |  |
| 18-29 | 1376 (18.8) | 344 (18.8) | 1 (ref) | 1 (ref) | 3720 (18.9) | 930 (18.9) | 1 (ref) | 1 (ref) | 1980 (22.0) | 495 (22.0) | 1 (ref) | 1 (ref) |
| 30-39 | 832 (11.4) | 208 (11.4) | 1.00 (1.00-1.00) | 0.80 (0.75-0.86) | 3448 (17.6) | 862 (17.6) | 1.00 (1.00-1.00) | 0.74 (0.71-0.76) | 1912 (21.2) | 478 (21.2) | 1.00 (1.00-1.00) | 0.85 (0.82-0.88) |
| 40-49 | 2256 (30.9) | 564 (30.9) | 1.00 (1.00-1.00) | 0.81 (0.75-0.87) | 5972 (30.4) | 1493 (30.4) | 1.00 (1.00-1.00) | 0.76 (0.74-0.79) | 3060 (34.0) | 765 (34.0) | 1.00 (1.00-1.00) | 1.02 (0.98-1.08) |
| 50-59 | 1656 (22.7) | 414 (22.7) | 1.00 (1.00-1.00) | 1.04 (0.98-1.10) | 4088 (20.8) | 1022 (20.8) | 1.00 (1.00-1.00) | 0.91 (0.88-0.94) | 1512 (16.8) | 378 (16.8) | 1.00 (1.00-1.00) | 1.23 (1.15-1.29) |
| 60-69 | 568 (7.8) | 142 (7.8) | 1.00 (1.00-1.00) | 1.74 (1.57-1.91) | 1348 (6.9) | 337 (6.9) | 1.00 (1.00-1.00) | 1.33 (1.25-1.43) | 220 (2.4) | 55 (2.4) | 1.00 (1.00-1.00) | 3.86 (3.23-4.39) |
| 70+ | 620 (8.5) | 155 (8.5) | 1.00 (1.00-1.00) | 2.06 (1.77-2.32) | 1060 (5.4) | 265 (5.4) | 1.00 (1.00-1.00) | 1.46 (1.35-1.57) | 324 (3.6) | 81 (3.6) | 1.00 (1.00-1.00) | 6.33 (5.04-7.72) |
| **Population in the area of residence** |  |  |  |  |  |  |  |  |  |  |  |  |
| <5000 inhabitants | 2016 (27.6) | 504 (27.6) | 1 (ref) | 1 (ref) | 5096 (26.0) | 1274 (26.0) | 1 (ref) | 1 (ref) | 1972 (21.9) | 493 (21.9) | 1 (ref) | 1 (ref) |
| 5000 – 19,999 inhabitants | 668 (9.1) | 167 (9.1) | 1.00 (1.00-1.00) | 1.09 (1.04-1.13) | 1524 (7.8) | 381 (7.8) | 1.00 (1.00-1.00) | 1.01 (0.98-1.04) | 392 (4.4) | 98 (4.4) | 1.00 (1.00-1.00) | 1.06 (1.01-1.11) |
| 20,000 – 99,999 inhabitants | 1004 (13.7) | 251 (13.7) | 1.00 (1.00-1.00) | 1.10 (1.04-1.14) | 1976 (10.1) | 494 (10.1) | 1.00 (1.00-1.00) | 1.05 (1.03-1.08) | 568 (6.3) | 142 (6.3) | 1.00 (1.00-1.00) | 1.09 (1.04-1.14) |
| Over 100,000 inhabitants | 2712 (37.1) | 678 (37.1) | 1.00 (1.00-1.00) | 1.18 (1.14-1.24) | 6840 (34.8) | 1710 (34.8) | 1.00 (1.00-1.00) | 1.11 (1.09-1.14) | 2912 (32.3) | 728 (32.3) | 1.00 (1.00-1.00) | 1.08 (1.05-1.11) |
| Greater Paris area | 908 (12.4) | 227 (12.4) | 1.00 (1.00-1.00) | 1.23 (1.12-1.39) | 4200 (21.4) | 1050 (21.4) | 1.00 (1.00-1.00) | 1.08 (1.04-1.14) | 3164 (35.1) | 791 (35.1) | 1.00 (1.00-1.00) | 0.96 (0.90-1.03) |
| **Region of residence** |  |  |  |  |  |  |  |  |  |  |  |  |
| Ile-de-France | 1072 (14.7) | 268 (14.7) | 1 (ref) | 1 (ref) | 4684 (23.9) | 1171 (23.9) | 1 (ref) | 1 (ref) | 3376 (37.5) | 844 (37.5) | 1 (ref) | 1 (ref) |
| Auverge-Rhône-Alpes | 1072 (14.7) | 268 (14.7) | 1.00 (1.00-1.00) | 0.98 (0.89-1.08) | 3276 (16.7) | 819 (16.7) | 1.00 (1.00-1.00) | 0.98 (0.93-1.01) | 1296 (14.4) | 324 (14.4) | 1.00 (1.00-1.00) | 0.94 (0.88-0.99) |
| Occitanie | 380 (5.2) | 95 (5.2) | 1.00 (1.00-1.00) | 0.93 (0.83-1.02) | 1356 (6.9) | 339 (6.9) | 1.00 (1.00-1.00) | 0.96 (0.91-1.00) | 532 (5.9) | 133 (5.9) | 1.00 (1.00-1.00) | 0.94 (0.87-1.02) |
| Provence-Alpes-Côte d'Azur and Corsica | 484 (6.6) | 121 (6.6) | 1.00 (1.00-1.00) | 0.98 (0.89-1.08) | 2104 (10.7) | 526 (10.7) | 1.00 (1.00-1.00) | 0.95 (0.91-0.98) | 588 (6.5) | 147 (6.5) | 1.00 (1.00-1.00) | 0.94 (0.88-1.02) |
| Grand Est | 1336 (18.3) | 334 (18.3) | 1.00 (1.00-1.00) | 0.95 (0.87-1.05) | 2020 (10.3) | 505 (10.3) | 1.00 (1.00-1.00) | 0.94 (0.89-0.98) | 420 (4.7) | 105 (4.7) | 1.00 (1.00-1.00) | 1.05 (0.98-1.12) |
| Nouvelle-Aquitaine | 360 (4.9) | 90 (4.9) | 1.00 (1.00-1.00) | 0.91 (0.82-1.03) | 1144 (5.8) | 286 (5.8) | 1.00 (1.00-1.00) | 0.96 (0.92-1.01) | 520 (5.8) | 130 (5.8) | 1.00 (1.00-1.00) | 0.99 (0.93-1.06) |
| Hauts-de-France | 572 (7.8) | 143 (7.8) | 1.00 (1.00-1.00) | 0.95 (0.87-1.10) | 1828 (9.3) | 457 (9.3) | 1.00 (1.00-1.00) | 0.93 (0.89-0.97) | 824 (9.1) | 206 (9.1) | 1.00 (1.00-1.00) | 0.94 (0.88-1.00) |
| Pays de la Loire | 392 (5.4) | 98 (5.4) | 1.00 (1.00-1.00) | 0.90 (0.80-0.99) | 700 (3.6) | 175 (3.6) | 1.00 (1.00-1.00) | 0.94 (0.89-0.98) | 368 (4.1) | 92 (4.1) | 1.00 (1.00-1.00) | 0.97 (0.90-1.04) |
| Bretagne | 244 (3.3) | 61 (3.3) | 1.00 (1.00-1.00) | 0.85 (0.78-0.96) | 404 (2.1) | 101 (2.1) | 1.00 (1.00-1.00) | 0.94 (0.89-1.00) | 236 (2.6) | 59 (2.6) | 1.00 (1.00-1.00) | 0.91 (0.86-0.99) |
| Normandie | 360 (4.9) | 90 (4.9) | 1.00 (1.00-1.00) | 0.94 (0.85-1.08) | 740 (3.8) | 185 (3.8) | 1.00 (1.00-1.00) | 0.96 (0.91-1.00) | 316 (3.5) | 79 (3.5) | 1.00 (1.00-1.00) | 0.99 (0.93-1.05) |
| Bourgogne-Franche-Comté | 828 (11.3) | 207 (11.3) | 1.00 (1.00-1.00) | 0.99 (0.91-1.10) | 848 (4.3) | 212 (4.3) | 1.00 (1.00-1.00) | 0.95 (0.91-1.00) | 276 (3.1) | 69 (3.1) | 1.00 (1.00-1.00) | 0.94 (0.86-1.02) |
| Centre-Val de Loire | 208 (2.8) | 52 (2.8) | 1.00 (1.00-1.00) | 0.97 (0.85-1.12) | 532 (2.7) | 133 (2.7) | 1.00 (1.00-1.00) | 0.94 (0.90-0.99) | 256 (2.8) | 64 (2.8) | 1.00 (1.00-1.00) | 0.91 (0.86-0.98) |
| **Education level** |  |  |  |  |  |  |  |  |  |  |  |  |
| No diploma | 32 (0.4) | 7 (0.4) | - | - | 523 (2.7) | 66 (1.3) | 1 (ref) | 1 (ref) | 279 (3.1) | 39 (1.7) | 1 (ref) | 1 (ref) |
| Pre-high school diploma | 223 (3.0) | 54 (3.0) | - | - | 3434 (17.5) | 791 (16.1) | 0.55 (0.51-0.59) | 0.52 (0.48-0.57) | 1393 (15.5) | 289 (12.8) | 0.67 (0.63-0.74) | 0.65 (0.59-0.73) |
| High-school diploma | 242 (3.3) | 76 (4.2) | - | - | 4168 (21.2) | 1166 (23.8) | 0.45 (0.42-0.49) | 0.43 (0.39-0.48) | 1893 (21.0) | 553 (24.6) | 0.48 (0.45-0.52) | 0.46 (0.41-0.52) |
| Bachelor's degree | 425 (5.8) | 152 (8.3) | - | - | 7250 (36.9) | 2016 (41.1) | 0.46 (0.42-0.49) | 0.38 (0.34-0.42) | 3207 (35.6) | 916 (40.7) | 0.49 (0.45-0.55) | 0.42 (0.37-0.47) |
| Master's degree or higher | 174 (2.4) | 60 (3.3) | - | - | 4261 (21.7) | 870 (17.7) | 0.62 (0.58-0.66) | 0.47 (0.42-0.52) | 2236 (24.8) | 455 (20.2) | 0.69 (0.64-0.75) | 0.55 (0.47-0.62) |
| Missing | 6213 (85.0) | 1478 (80.9) | - | - |  |  |  |  |  |  |  |  |
| **Health conditions** |  |  |  |  |  |  |  |  |  |  |  |  |
| Diabetes mellitus | 259 (3.5) | 97 (5.3) | 0.66 (0.58-0.72) | 0.70 (0.62-0.78) | 572 (2.9) | 185 (3.8) | 0.77 (0.72-0.81) | 0.74 (0.69-0.79) | 177 (2.0) | 64 (2.8) | 0.69 (0.61-0.75) | 0.76 (0.68-0.85) |
| Hypertension | 795 (10.9) | 211 (11.5) | 0.93 (0.88-1.00) | 1.02 (0.96-1.12) | 1741 (8.9) | 462 (9.4) | 0.93 (0.90-0.97) | 0.94 (0.90-1.00) | 607 (6.7) | 188 (8.3) | 0.79 (0.75-0.84) | 0.85 (0.79-0.91) |
| Chronic respiratory disease | 642 (8.8) | 146 (8) | 1.11 (1.01-1.19) | 1.19 (1.10-1.31) | 1542 (7.9) | 345 (7.0) | 1.13 (1.08-1.17) | 1.24 (1.18-1.28) | 751 (8.3) | 164 (7.3) | 1.16 (1.08-1.23) | 1.22 (1.14-1.31) |
| Coronary artery disease | 85 (1.2) | 16 (0.9) | 1.32 (1.11-1.60) | 1.40 (1.10-1.66) | 182 (0.9) | 32 (0.7) | 1.43 (1.27-1.58) | 1.52 (1.33-1.73) | 30 (0.3) | 8 (0.4) | 0.94 (0.66-1.19) | 1.28 (0.74-1.70) |
| **Body-mass index (kg/m²)** |  |  |  |  |  |  |  |  |  |  |  |  |
| Healthy weight (≥18.5 & <25) | 3713 (50.8) | 906 (49.6) | 1 (ref) | 1 (ref) | 10221 (52.1) | 2530 (51.5) | 1 (ref) | 1 (ref) | 4759 (52.8) | 1160 (51.5) | 1 (ref) | 1 (ref) |
| Underweight (<18.5) | 217 (3.0) | 75 (4.1) | 0.70 (0.61-0.81) | 0.77 (0.68-0.89) | 642 (3.3) | 227 (4.6) | 0.70 (0.65-0.74) | 0.71 (0.66-0.77) | 280 (3.1) | 120 (5.3) | 0.57 (0.52-0.61) | 0.57 (0.51-0.62) |
| Overweight (≥25 & <30) | 2184 (29.9) | 524 (28.7) | 1.02 (0.97-1.07) | 1.04 (0.99-1.10) | 5619 (28.6) | 1369 (27.9) | 1.02 (0.99-1.04) | 1.03 (0.99-1.06) | 2547 (28.3) | 572 (25.4) | 1.08 (1.04-1.12) | 1.12 (1.06-1.17) |
| Obesity (≥30) | 1194 (16.3) | 322 (17.6) | 0.91 (0.85-0.96) | 0.99 (0.93-1.05) | 3153 (16.1) | 783 (16) | 1.00 (0.96-1.03) | 1.03 (0.99-1.07) | 1422 (15.8) | 400 (17.8) | 0.87 (0.82-0.90) | 0.92 (0.86-0.98) |
| **Housing** |  |  |  |  |  |  |  |  |  |  |  |  |
| Individual house | 4652 (63.7) | 1062 (58.1) | 1 (ref) | 1 (ref) | 11617 (59.2) | 2752 (56.1) | 1 (ref) | 1 (ref) | 4656 (51.7) | 1142 (50.7) | 1 (ref) | 1 (ref) |
| Apartment | 2612 (35.7) | 756 (41.4) | 0.79 (0.76-0.82) | 0.95 (0.89-1.02) | 7867 (40.1) | 2135 (43.5) | 0.87 (0.86-0.89) | 1.02 (0.99-1.06) | 4291 (47.6) | 1104 (49) | 0.95 (0.93-0.97) | 1.05 (1.00-1.10) |
| Shelter | 39 (0.5) | 8 (0.4) | 1.10 (0.88-1.41) | 1.58 (1.15-2.20) | 122 (0.6) | 20 (0.4) | 1.45 (1.24-1.67) | 1.85 (1.45-2.27) | 52 (0.6) | 6 (0.3) | 2.13 (1.67-2.63) | 2.75 (2.06-4.24) |
| Nursing home | 5 (0.1) | 1 (0.1) | 1.14 (0.45-2.07) | 0.77 (0.26-1.70) | 30 (0.2) | 2 (0) | 3.65 (2.71-4.38) | 1.61 (1.00-2.45) | 9 (0.1) | 0 (0) | 1.00 (1.00-1.00) | 1.00 (1.00-1.00) |
| **Living with a child** |  |  |  |  |  |  |  |  |  |  |  |  |
| Attending daycare | 160 (2.2) | 27 (1.5) | 1.50 (1.28-1.71) | 1.38 (1.13-1.68) | 625 (3.2) | 122 (2.5) | 1.29 (1.21-1.37) | 1.19 (1.1-1.31) | 327 (3.6) | 58 (2.6) | 1.42 (1.31-1.55) | 1.32 (1.16-1.51) |
| Attended for by a professional in-home caregiver | 216 (3.0) | 25 (1.4) | 2.18 (1.92-2.55) | 1.81 (1.53-2.18) | 794 (4.0) | 111 (2.3) | 1.82 (1.70-1.94) | 1.61 (1.50-1.73) | 400 (4.4) | 58 (2.6) | 1.76 (1.61-1.89) | 1.58 (1.43-1.75) |
| Attending reschool | 557 (7.6) | 103 (5.6) | 1.38 (1.26-1.48) | 1.21 (1.09-1.39) | 2117 (10.8) | 435 (8.9) | 1.24 (1.20-1.30) | 1.11 (1.05-1.17) | 1204 (13.4) | 216 (9.6) | 1.45 (1.39-1.51) | 1.34 (1.26-1.45) |
| Attending primary school | 1102 (15.1) | 258 (14.1) | 1.08 (1.03-1.14) | 0.88 (0.81-0.96) | 3629 (18.5) | 763 (15.5) | 1.23 (1.20-1.26) | 1.14 (1.08-1.19) | 1935 (21.5) | 391 (17.4) | 1.31 (1.24-1.34) | 1.20 (1.10-1.26) |
| Attending middle school | 1303 (17.8) | 244 (13.4) | 1.41 (1.34-1.51) | 1.27 (1.15-1.39) | 3561 (18.1) | 755 (15.4) | 1.22 (1.19-1.26) | 1.12 (1.06-1.16) | 1742 (19.3) | 398 (17.7) | 1.12 (1.08-1.15) | 1.03 (0.98-1.09) |
| Attending high school | 1249 (17.1) | 252 (13.8) | 1.29 (1.22-1.38) | 1.15 (1.04-1.27) | 3165 (16.1) | 654 (13.3) | 1.25 (1.22-1.29) | 1.23 (1.17-1.27) | 1364 (15.1) | 353 (15.7) | 0.96 (0.92-0.99) | 0.94 (0.90-1.00) |
| Attending university | 867 (11.9) | 202 (11.1) | 1.09 (1.01-1.13) | 0.97 (0.88-1.07) | 1933 (9.8) | 536 (10.9) | 0.89 (0.86-0.92) | 0.85 (0.81-0.90) | 903 (10.0) | 254 (11.3) | 0.88 (0.84-0.93) | 0.88 (0.82-0.94) |
| **Number of people in the household** |  |  |  |  |  |  |  |  |  |  |  |  |
| 1 | 1236 (16.9) | 395 (21.6) | 1 (ref) | 1 (ref) | 3231 (16.5) | 947 (19.3) | 1 (ref) | 1 (ref) | 1434 (15.9) | 439 (19.5) | 1 (ref) | 1 (ref) |
| 2 | 2314 (31.7) | 629 (34.4) | 1.18 (1.09-1.25) | 1.07 (0.99-1.15) | 5571 (28.4) | 1538 (31.3) | 1.06 (1.02-1.11) | 1.00 (0.96-1.04) | 2274 (25.2) | 577 (25.6) | 1.21 (1.15-1.28) | 1.20 (1.11-1.29) |
| 3 | 1419 (19.4) | 354 (19.4) | 1.28 (1.17-1.36) | 1.22 (1.09-1.33) | 4101 (20.9) | 1016 (20.7) | 1.19 (1.13-1.23) | 1.07 (1.00-1.12) | 1875 (20.8) | 515 (22.9) | 1.11 (1.05-1.17) | 1.04 (0.93-1.13) |
| 4 | 1601 (21.9) | 323 (17.7) | 1.58 (1.48-1.67) | 1.43 (1.25-1.57) | 4532 (23.1) | 1017 (20.7) | 1.31 (1.25-1.37) | 1.08 (1.02-1.15) | 2268 (25.2) | 479 (21.3) | 1.45 (1.38-1.53) | 1.35 (1.23-1.49) |
| 5 | 541 (7.4) | 99 (5.4) | 1.75 (1.59-1.89) | 1.63 (1.39-1.93) | 1631 (8.3) | 307 (6.3) | 1.56 (1.48-1.63) | 1.29 (1.20-1.43) | 830 (9.2) | 189 (8.4) | 1.35 (1.26-1.43) | 1.23 (1.09-1.41) |
| 6+ | 197 (2.7) | 27 (1.5) | 2.33 (2.05-2.71) | 2.29 (1.79-2.75) | 570 (2.9) | 84 (1.7) | 1.99 (1.83-2.14) | 1.55 (1.41-1.74) | 328 (3.6) | 53 (2.4) | 1.90 (1.70-2.07) | 1.75 (1.52-2.13) |
| **Workplace** |  |  |  |  |  |  |  |  |  |  |  |  |
| No remote office, no open space | 809 (11.1) | 181 (9.9) | 1 (ref) | 1 (ref) | 1986 (10.1) | 489 (10) | 1 (ref) | 1 (ref) | 867 (9.6) | 203 (9.0) | 1 (ref) | 1 (ref) |
| No remote office, open space | 461 (6.3) | 84 (4.6) | 1.22 (1.11-1.41) | 1.33 (1.18-1.54) | 1080 (5.5) | 263 (5.4) | 1.01 (0.94-1.08) | 1.12 (1.05-1.2) | 563 (6.2) | 95 (4.2) | 1.38 (1.29-1.54) | 1.36 (1.24-1.50) |
| Not working | 1617 (22.1) | 538 (29.4) | 0.67 (0.63-0.73) | 0.60 (0.53-0.68) | 3887 (19.8) | 1263 (25.7) | 0.76 (0.73-0.79) | 0.70 (0.66-0.75) | 1454 (16.1) | 475 (21.1) | 0.72 (0.67-0.77) | 0.83 (0.74-0.89) |
| Working in a non-office setting | 2892 (39.6) | 597 (32.7) | 1.09 (1.01-1.15) | 0.97 (0.88-1.06) | 8026 (40.9) | 1650 (33.6) | 1.2 (1.15-1.26) | 1.04 (0.99-1.10) | 3344 (37.1) | 809 (35.9) | 0.97 (0.92-1.03) | 0.90 (0.83-0.96) |
| Partial remote office, no open space | 489 (6.7) | 132 (7.2) | 0.83 (0.72-0.92) | 0.77 (0.69-0.86) | 1501 (7.6) | 421 (8.6) | 0.88 (0.83-0.93) | 0.86 (0.80-0.91) | 832 (9.2) | 230 (10.2) | 0.85 (0.78-0.91) | 0.76 (0.68-0.83) |
| Partial remote office, open space | 379 (5.2) | 90 (4.9) | 0.94 (0.85-1.04) | 0.89 (0.79-1.06) | 1020 (5.2) | 341 (6.9) | 0.74 (0.68-0.79) | 0.72 (0.67-0.78) | 651 (7.2) | 182 (8.1) | 0.84 (0.77-0.91) | 0.74 (0.65-0.81) |
| Complete remote office | 661 (9.0) | 205 (11.2) | 0.72 (0.65-0.78) | 0.67 (0.60-0.77) | 2136 (10.9) | 482 (9.8) | 1.09 (1.03-1.15) | 0.95 (0.89-1.00) | 1298 (14.4) | 258 (11.5) | 1.18 (1.12-1.26) | 0.84 (0.78-0.90) |
| On leave during the period of interest |  |  |  |  |  |  |  |  |  |  |  |  |
| **Gatherings** |  |  |  |  |  |  |  |  |  |  |  |  |
| Professional meeting | 2121 (29.0) | 410 (22.4) | 1.42 (1.35-1.47) | 1.14 (1.07-1.19) | 4820 (24.5) | 1184 (24.1) | 1.02 (0.99-1.06) | 1.01 (0.97-1.05) | 2053 (22.8) | 546 (24.2) | 0.92 (0.89-0.96) | 0.96 (0.92-1.02) |
| Private meeting | 3280 (44.9) | 598 (32.7) | 1.67 (1.61-1.73) | 1.75 (1.67-1.85) | 9287 (47.3) | 2551 (52.0) | 0.83 (0.81-0.85) | 0.91 (0.88-0.93) | 4130 (45.8) | 1183 (52.5) | 0.76 (0.74-0.79) | 0.84 (0.81-0.88) |
| Religious gathering | 222 (3.0) | 46 (2.5) | 1.21 (1.07-1.40) | 1.01 (0.88-1.15) | 596 (3.0) | 162 (3.3) | 0.91 (0.86-0.98) | 1.00 (0.92-1.08) | 307 (3.4) | 92 (4.1) | 0.83 (0.76-0.91) | 1.00 (0.90-1.12) |
| **Retail, shops** |  |  |  |  |  |  |  |  |  |  |  |  |
| Any shop | 5864 (80.2) | 1498 (82.0) | 0.89 (0.85-0.94) | 0.83 (0.79-0.88) | 13460 (68.5) | 3951 (80.5) | 0.53 (0.51-0.54) | 0.56 (0.54-0.57) |  |  |  |  |
| Mall |  |  |  |  |  |  |  |  | 535 (5.9) | 244 (10.8) | 0.52 (0.49-0.55) | 0.66 (0.61-0.73) |
| Market |  |  |  |  |  |  |  |  | 964 (10.7) | 344 (15.3) | 0.66 (0.63-0.70) | 0.84 (0.78-0.90) |
| Convenience store |  |  |  |  |  |  |  |  | 2461 (27.3) | 798 (35.4) | 0.69 (0.66-0.71) | 0.92 (0.86-0.96) |
| Supermarket |  |  |  |  |  |  |  |  | 3999 (44.4) | 1392 (61.8) | 0.49 (0.48-0.51) | 0.57 (0.55-0.59) |
| Other shop |  |  |  |  |  |  |  |  | 231 (2.6) | 107 (4.8) | 0.53 (0.48-0.57) | 0.62 (0.54-0.68) |
| Hairdresser |  |  |  |  |  |  |  |  | 668 (7.4) | 336 (14.9) | 0.46 (0.43-0.48) | 0.52 (0.48-0.55) |
| Take-away delivery |  |  |  |  |  |  |  |  | 3667 (40.7) | 993 (44.1) | 0.87 (0.84-0.90) | 0.97 (0.92-1.01) |
| Home delivery |  |  |  |  |  |  |  |  | 2797 (31.1) | 673 (29.9) | 1.06 (1.02-1.09) | 1.13 (1.08-1.19) |
| **Shared transport** |  |  |  |  |  |  |  |  |  |  |  |  |
| Metro | 549 (7.5) | 164 (9.0) | 0.83 (0.77-0.88) | 0.92 (0.82-1.02) | 1774 (9.0) | 583 (11.9) | 0.74 (0.71-0.77) | 1.00 (0.94-1.05) | 1337 (14.8) | 376 (16.7) | 0.87 (0.83-0.91) | 1.19 (1.12-1.28) |
| Tram | 309 (4.2) | 126 (6.9) | 0.59 (0.54-0.65) | 0.72 (0.63-0.80) | 794 (4.0) | 338 (6.9) | 0.57 (0.54-0.60) | 0.77 (0.72-0.83) | 487 (5.4) | 182 (8.1) | 0.65 (0.60-0.70) | 0.88 (0.79-0.95) |
| Short-distance bus | 544 (7.4) | 227 (12.4) | 0.57 (0.52-0.61) | 0.65 (0.58-0.72) | 1644 (8.4) | 716 (14.6) | 0.53 (0.51-0.56) | 0.65 (0.61-0.68) | 1013 (11.2) | 387 (17.2) | 0.61 (0.58-0.64) | 0.63 (0.59-0.68) |
| Short-distance train | 378 (5.2) | 101 (5.5) | 0.93 (0.84-1.03) | 0.91 (0.76-1.03) | 950 (4.8) | 393 (8.0) | 0.59 (0.55-0.61) | 0.64 (0.60-0.70) | 479 (5.3) | 232 (10.3) | 0.49 (0.45-0.53) | 0.46 (0.41-0.50) |
| Airplane travel | 91 (1.2) | 19 (1.0) | 1.21 (1.00-1.43) | 0.69 (0.50-0.89) | 230 (1.2) | 55 (1.1) | 1.04 (0.91-1.16) | 1.78 (1.51-2.12) | 115 (1.3) | 27 (1.2) | 1.07 (0.92-1.22) | 1.35 (1.10-1.73) |
| Boat travel | 9 (0.1) | 2 (0.1) | 1.06 (0.37-2.00) | 0.58 (0.13-1.20) | 9 (0) | 3 (0.1) | 0.75 (0.33-1.17) | 0.83 (0.34-1.71) | 10 (0.1) | 3 (0.1) | 0.87 (0.42-1.33) | 0.98 (0.39-1.68) |
| Long-distance bus travel | 38 (0.5) | 8 (0.4) | 1.16 (0.87-1.63) | 1.23 (0.78-1.83) | 71 (0.4) | 43 (0.9) | 0.41 (0.32-0.50) | 0.64 (0.51-0.86) | 34 (0.4) | 21 (0.9) | 0.39 (0.31-0.52) | 0.70 (0.52-0.92) |
| Long-distance train travel | 245 (3.3) | 48 (2.6) | 1.28 (1.16-1.44) | 1.41 (1.14-1.81) | 648 (3.3) | 180 (3.7) | 0.90 (0.84-0.96) | 1.86 (1.72-2.06) | 321 (3.6) | 97 (4.3) | 0.82 (0.76-0.88) | 1.88 (1.63-2.17) |
| Car-pooling | 654 (8.9) | 107 (5.9) | 1.58 (1.47-1.68) | 1.47 (1.35-1.60) | 1753 (8.9) | 298 (6.1) | 1.51 (1.46-1.57) | 1.74 (1.68-1.85) | 1929 (21.4) | 523 (23.2) | 0.90 (0.87-0.93) | 1.06 (1.02-1.11) |
| Taxi |  |  |  |  |  |  |  |  | 393 (4.4) | 99 (4.4) | 0.99 (0.90-1.08) | 1.29 (1.08-1.39) |
| **Leisure activities** |  |  |  |  |  |  |  |  |  |  |  |  |
| Any cultural venue | 403 (5.5) | 85 (4.7) | 1.19 (1.09-1.33) | 0.94 (0.83-1.05) | 53 (0.3) | 31 (0.6) | 0.41 (0.34-0.53) | 0.61 (0.49-0.81) |  |  |  |  |
| Indoor sports practice | 382 (5.2) | 65 (3.6) | 1.50 (1.36-1.62) | 1.23 (1.11-1.36) |  |  |  |  | 73 (0.8) | 30 (1.3) | 0.60 (0.49-0.71) | 1.19 (0.89-1.47) |
| Swimming pool | 137 (1.9) | 32 (1.8) | 1.07 (0.88-1.22) | 0.87 (0.72-1.07) |  |  |  |  | 62 (0.7) | 31 (1.4) | 0.50 (0.42-0.59) | 0.58 (0.48-0.70) |
| Outdoor sports practice |  |  |  |  |  |  |  |  | 2395 (26.6) | 758 (33.7) | 0.72 (0.69-0.74) | 0.72 (0.69-0.75) |
| Bar or restaurant | 1902 (26.0) | 286 (15.7) | 1.90 (1.81-1.97) | 1.97 (1.84-2.07) |  |  |  |  |  |  |  |  |
| Party | 153 (2.1) | 27 (1.5) | 1.43 (1.23-1.63) | 1.15 (0.90-1.41) | 196 (1.0) | 50 (1.0) | 0.98 (0.85-1.13) | 1.66 (1.42-1.94) | 136 (1.5) | 30 (1.3) | 1.14 (1.00-1.30) | 1.84 (1.52-2.20) |

Legend: Adjusted odds ratios estimated in models adjusted for all variables shown in the table, as well as week of exposure, health status (diabetes, hypertension, respiratory disease, coronary artery disease, immunosuppressive treatment, body-mass index categorized as <18.5 kg/m², [18.5-25[, [25-30[, and ≥30 kg/m²), COVID-19 vaccine status (categorized as number of doses and time since last dose as <90 days, 90-179 days, ≥180 days, with a specific category for participants with missing data for the date of the last dose), past SARS-CoV-2 infection (categorized as 61-180 days prior or over 180 days prior), smoking status, level of education, and professional category (of the person in the household with the highest income for the first two periods, of the participant for the following periods)). Empty cells reflect changes of the questionnaire or periods when specific settings were closed.Table S2: Exposure prevalence, odds ratios of SARS-CoV-2 infection in univariable and multivariable estimates in a case-control study conducted in mainland France (2020-2022) – Results for the fourth, fifth, and sixth periods of the study (June 14 to December 19, 2021)

| Period (onset date) | 4: 06/14/2021 | | | | 5: 08/14/2021 | | | | 6: 10/02/2021 | | | |
| --- | --- | --- | --- | --- | --- | --- | --- | --- | --- | --- | --- | --- |
|  | Cases | Controls | OR (95% CI) | Adjusted OR (95% CI) | Cases | Controls | OR (95% CI) | Adjusted OR (95% CI) | Cases | Controls | OR (95% CI) | Adjusted OR (95% CI) |
| Male sex | 2744 (24.4) | 686 (24.4) | 1.00 (1.00-1.00) | 1.08 (1.04-1.12) | 1316 (27.3) | 329 (27.3) | 1.00 (1.00-1.00) | 0.99 (0.95-1.05) | 3756 (33.4) | 939 (33.4) | 1.00 (1.00-1.00) | 1.11 (1.08-1.15) |
| **Age (years)** |  |  |  |  |  |  |  |  |  |  |  |  |
| 18-29 | 3212 (28.5) | 803 (28.5) | 1 (ref) | 1 (ref) | 1104 (22.9) | 276 (22.9) | 1 (ref) | 1 (ref) | 1108 (9.9) | 277 (9.9) | 1 (ref) | 1 (ref) |
| 30-39 | 2172 (19.3) | 543 (19.3) | 1.00 (1.00-1.00) | 1.12 (1.07-1.18) | 888 (18.4) | 222 (18.4) | 1.00 (1.00-1.00) | 0.76 (0.71-0.83) | 2044 (18.2) | 511 (18.2) | 1.00 (1.00-1.00) | 0.84 (0.80-0.90) |
| 40-49 | 3472 (30.8) | 868 (30.8) | 1.00 (1.00-1.00) | 1.44 (1.37-1.52) | 1528 (31.7) | 382 (31.7) | 1.00 (1.00-1.00) | 0.84 (0.79-0.92) | 3316 (29.5) | 829 (29.5) | 1.00 (1.00-1.00) | 0.92 (0.87-0.98) |
| 50-59 | 1900 (16.9) | 475 (16.9) | 1.00 (1.00-1.00) | 1.73 (1.64-1.83) | 908 (18.8) | 227 (18.8) | 1.00 (1.00-1.00) | 1.21 (1.12-1.32) | 2408 (21.4) | 602 (21.4) | 1.00 (1.00-1.00) | 1.15 (1.09-1.25) |
| 60-69 | 348 (3.1) | 87 (3.1) | 1.00 (1.00-1.00) | 2.79 (2.52-3.22) | 132 (2.7) | 33 (2.7) | 1.00 (1.00-1.00) | 2.91 (2.11-3.95) | 1828 (16.3) | 457 (16.3) | 1.00 (1.00-1.00) | 1.38 (1.27-1.52) |
| 70+ | 160 (1.4) | 40 (1.4) | 1.00 (1.00-1.00) | 2.51 (2.11-3.06) | 260 (5.4) | 65 (5.4) | 1.00 (1.00-1.00) | 2.63 (1.92-3.58) | 544 (4.8) | 136 (4.8) | 1.00 (1.00-1.00) | 1.63 (1.46-1.81) |
| **Population in the area of residence** |  |  |  |  |  |  |  |  |  |  |  |  |
| <5000 inhabitants | 1796 (15.9) | 449 (15.9) | 1 (ref) | 1 (ref) | 692 (14.4) | 173 (14.4) | 1 (ref) | 1 (ref) | 2528 (22.5) | 632 (22.5) | 1 (ref) | 1 (ref) |
| 5000 – 19,999 inhabitants | 548 (4.9) | 137 (4.9) | 1.00 (1.00-1.00) | 0.99 (0.94-1.03) | 176 (3.7) | 44 (3.7) | 1.00 (1.00-1.00) | 0.98 (0.90-1.05) | 620 (5.5) | 155 (5.5) | 1.00 (1.00-1.00) | 0.98 (0.94-1.02) |
| 20,000 – 99,999 inhabitants | 668 (5.9) | 167 (5.9) | 1.00 (1.00-1.00) | 1.03 (0.99-1.07) | 292 (6.1) | 73 (6.1) | 1.00 (1.00-1.00) | 0.99 (0.93-1.06) | 868 (7.7) | 217 (7.7) | 1.00 (1.00-1.00) | 1.02 (0.99-1.06) |
| Over 100,000 inhabitants | 4744 (42.1) | 1186 (42.1) | 1.00 (1.00-1.00) | 0.98 (0.95-1.02) | 2180 (45.2) | 545 (45.2) | 1.00 (1.00-1.00) | 1.05 (1.00-1.11) | 4104 (36.5) | 1026 (36.5) | 1.00 (1.00-1.00) | 1.03 (1.00-1.06) |
| Greater Paris area | 3508 (31.1) | 877 (31.1) | 1.00 (1.00-1.00) | 0.87 (0.82-0.93) | 1480 (30.7) | 370 (30.7) | 1.00 (1.00-1.00) | 0.88 (0.79-0.95) | 3128 (27.8) | 782 (27.8) | 1.00 (1.00-1.00) | 0.90 (0.82-1.00) |
| **Region of residence** |  |  |  |  |  |  |  |  |  |  |  |  |
| Ile-de-France | 3564 (31.6) | 891 (31.6) | 1 (ref) | 1 (ref) | 1500 (31.1) | 375 (31.1) | 1 (ref) | 1 (ref) | 3244 (28.8) | 811 (28.8) | 1 (ref) | 1 (ref) |
| Auverge-Rhône-Alpes | 1284 (11.4) | 321 (11.4) | 1.00 (1.00-1.00) | 1.01 (0.95-1.08) | 660 (13.7) | 165 (13.7) | 1.00 (1.00-1.00) | 0.81 (0.75-0.88) | 1728 (15.4) | 432 (15.4) | 1.00 (1.00-1.00) | 0.97 (0.89-1.06) |
| Occitanie | 1880 (16.7) | 470 (16.7) | 1.00 (1.00-1.00) | 0.92 (0.86-0.99) | 672 (13.9) | 168 (13.9) | 1.00 (1.00-1.00) | 0.81 (0.73-0.87) | 932 (8.3) | 233 (8.3) | 1.00 (1.00-1.00) | 0.96 (0.86-1.05) |
| Provence-Alpes-Côte d'Azur and Corsica | 1388 (12.3) | 347 (12.3) | 1.00 (1.00-1.00) | 0.92 (0.85-0.98) | 1032 (21.4) | 258 (21.4) | 1.00 (1.00-1.00) | 0.76 (0.67-0.82) | 1072 (9.5) | 268 (9.5) | 1.00 (1.00-1.00) | 0.94 (0.86-1.03) |
| Grand Est | 524 (4.7) | 131 (4.7) | 1.00 (1.00-1.00) | 1.04 (0.97-1.11) | 232 (4.8) | 58 (4.8) | 1.00 (1.00-1.00) | 0.85 (0.76-0.94) | 840 (7.5) | 210 (7.5) | 1.00 (1.00-1.00) | 1.04 (0.94-1.14) |
| Nouvelle-Aquitaine | 1208 (10.7) | 302 (10.7) | 1.00 (1.00-1.00) | 1.04 (0.97-1.12) | 300 (6.2) | 75 (6.2) | 1.00 (1.00-1.00) | 0.87 (0.78-0.96) | 976 (8.7) | 244 (8.7) | 1.00 (1.00-1.00) | 1.07 (0.97-1.16) |
| Hauts-de-France | 396 (3.5) | 99 (3.5) | 1.00 (1.00-1.00) | 1.08 (1.00-1.17) | 232 (4.8) | 58 (4.8) | 1.00 (1.00-1.00) | 0.94 (0.84-1.04) | 828 (7.4) | 207 (7.4) | 1.00 (1.00-1.00) | 1.02 (0.93-1.12) |
| Pays de la Loire | 356 (3.2) | 89 (3.2) | 1.00 (1.00-1.00) | 1.04 (0.95-1.14) | 76 (1.6) | 19 (1.6) | 1.00 (1.00-1.00) | 1.21 (1.07-1.35) | 636 (5.7) | 159 (5.7) | 1.00 (1.00-1.00) | 0.98 (0.89-1.06) |
| Bretagne | 304 (2.7) | 76 (2.7) | 1.00 (1.00-1.00) | 0.96 (0.88-1.02) | 24 (0.5) | 6 (0.5) | 1.00 (1.00-1.00) | 0.89 (0.71-1.05) | 356 (3.2) | 89 (3.2) | 1.00 (1.00-1.00) | 0.94 (0.87-1.03) |
| Normandie | 256 (2.3) | 64 (2.3) | 1.00 (1.00-1.00) | 0.95 (0.87-1.02) | 44 (0.9) | 11 (0.9) | 1.00 (1.00-1.00) | 0.92 (0.80-1.02) | 240 (2.1) | 60 (2.1) | 1.00 (1.00-1.00) | 1.09 (0.98-1.20) |
| Bourgogne-Franche-Comté | 48 (0.4) | 12 (0.4) | 1.00 (1.00-1.00) | 1.23 (1.08-1.36) | 40 (0.8) | 10 (0.8) | 1.00 (1.00-1.00) | 1.02 (0.89-1.17) | 192 (1.7) | 48 (1.7) | 1.00 (1.00-1.00) | 1.01 (0.90-1.12) |
| Centre-Val de Loire | 56 (0.5) | 14 (0.5) | 1.00 (1.00-1.00) | 1.45 (1.32-1.59) | 8 (0.2) | 2 (0.2) | 1.00 (1.00-1.00) | 1.21 (0.96-1.53) | 204 (1.8) | 51 (1.8) | 1.00 (1.00-1.00) | 0.84 (0.76-0.93) |
| **Education level** |  |  |  |  |  |  |  |  |  |  |  |  |
| No diploma | 266 (2.4) | 38 (1.3) | 1 (ref) | 1 (ref) | 152 (3.2) | 19 (1.6) | 1 (ref) | 1 (ref) | 204 (1.8) | 50 (1.8) | 1 (ref) | 1 (ref) |
| Pre-high school diploma | 1339 (11.9) | 310 (11) | 0.62 (0.56-0.69) | 0.60 (0.51-0.69) | 666 (13.8) | 173 (14.4) | 0.48 (0.42-0.55) | 0.52 (0.45-0.61) | 1670 (14.8) | 445 (15.8) | 0.92 (0.81-1.02) | 0.90 (0.77-1.01) |
| High-school diploma | 2179 (19.3) | 692 (24.6) | 0.45 (0.41-0.49) | 0.44 (0.37-0.50) | 961 (19.9) | 288 (23.9) | 0.42 (0.37-0.47) | 0.47 (0.40-0.54) | 1863 (16.6) | 656 (23.3) | 0.69 (0.62-0.78) | 0.69 (0.59-0.79) |
| Bachelor's degree | 4135 (36.7) | 1144 (40.6) | 0.51 (0.47-0.57) | 0.51 (0.43-0.59) | 1735 (36.0) | 455 (37.8) | 0.47 (0.41-0.53) | 0.53 (0.46-0.62) | 4128 (36.7) | 1123 (40.0) | 0.91 (0.79-1.00) | 0.78 (0.66-0.89) |
| Master's degree or higher | 3346 (29.7) | 632 (22.4) | 0.76 (0.69-0.83) | 0.71 (0.60-0.83) | 1306 (27.1) | 270 (22.4) | 0.61 (0.52-0.68) | 0.61 (0.51-0.71) | 3384 (30.1) | 538 (19.1) | 1.55 (1.36-1.69) | 1.18 (0.99-1.33) |
| **Health conditions** |  |  |  |  |  |  |  |  |  |  |  |  |
| Diabetes mellitus | 228 (2.0) | 71 (2.5) | 0.80 (0.72-0.88) | 0.89 (0.76-1.05) | 111 (2.3) | 43 (3.6) | 0.64 (0.54-0.72) | 0.67 (0.57-0.79) | 301 (2.7) | 152 (5.4) | 0.48 (0.44-0.53) | 0.56 (0.50-0.62) |
| Hypertension | 561 (5.0) | 182 (6.5) | 0.76 (0.71-0.81) | 0.82 (0.75-0.87) | 344 (7.1) | 109 (9.0) | 0.77 (0.71-0.84) | 0.76 (0.67-0.88) | 1163 (10.3) | 368 (13.1) | 0.76 (0.73-0.80) | 0.87 (0.82-0.93) |
| Chronic respiratory disease | 839 (7.5) | 176 (6.3) | 1.21 (1.15-1.27) | 1.18 (1.11-1.27) | 404 (8.4) | 73 (6.1) | 1.42 (1.30-1.52) | 1.58 (1.41-1.80) | 836 (7.4) | 184 (6.5) | 1.15 (1.07-1.21) | 1.33 (1.23-1.44) |
| Coronary artery disease | 72 (0.6) | 12 (0.4) | 1.50 (1.29-1.73) | 1.57 (1.12-1.96) | 31 (0.7) | 8 (0.7) | 0.97 (0.78-1.22) | 0.70 (0.54-0.9) | 104 (0.9) | 24 (0.9) | 1.08 (0.9-1.24) | 1.25 (1.01-1.48) |
| **Body-mass index (kg/m²)** |  |  |  |  |  |  |  |  |  |  |  |  |
| Healthy weight (≥18.5 & <25) | 6764 (60.1) | 1573 (55.9) | 1 (ref) | 1 (ref) | 2764 (57.4) | 595 (49.4) | 1 (ref) | 1 (ref) | 6170 (54.9) | 1375 (48.9) | 1 (ref) | 1 (ref) |
| Underweight (<18.5) | 544 (4.8) | 168 (6.0) | 0.75 (0.71-0.80) | 0.72 (0.66-0.78) | 218 (4.5) | 65 (5.4) | 0.72 (0.64-0.82) | 0.67 (0.58-0.78) | 402 (3.6) | 123 (4.4) | 0.73 (0.66-0.80) | 0.79 (0.72-0.88) |
| Overweight (≥25 & <30) | 2691 (23.9) | 678 (24.1) | 0.92 (0.89-0.95) | 1.05 (1.00-1.09) | 1231 (25.5) | 335 (27.8) | 0.79 (0.75-0.83) | 0.80 (0.74-0.85) | 3194 (28.4) | 816 (29) | 0.87 (0.84-0.90) | 0.97 (0.92-1.02) |
| Obesity (≥30) | 1265 (11.2) | 397 (14.1) | 0.74 (0.71-0.78) | 0.94 (0.87-0.99) | 607 (12.6) | 210 (17.4) | 0.62 (0.59-0.67) | 0.64 (0.59-0.70) | 1482 (13.2) | 498 (17.7) | 0.66 (0.63-0.70) | 0.76 (0.71-0.81) |
| **Housing** |  |  |  |  |  |  |  |  |  |  |  |  |
| Individual house | 4935 (43.8) | 1328 (47.2) | 1 (ref) | 1 (ref) | 2172 (45.1) | 544 (45.1) | 1 (ref) | 1 (ref) | 6406 (56.9) | 1579 (56.2) | 1 (ref) | 1 (ref) |
| Apartment | 6246 (55.5) | 1473 (52.3) | 1.14 (1.11-1.17) | 1.07 (1.02-1.13) | 2619 (54.3) | 658 (54.6) | 1.00 (0.97-1.03) | 1.11 (1.05-1.20) | 4803 (42.7) | 1228 (43.7) | 0.96 (0.95-0.98) | 1.06 (1.01-1.12) |
| Shelter | 82 (0.7) | 12 (0.4) | 1.84 (1.45-2.22) | 2.21 (1.69-3.05) | 24 (0.5) | 3 (0.2) | 2.02 (1.35-2.71) | 1.59 (0.93-2.38) | 37 (0.3) | 5 (0.2) | 1.82 (1.33-2.29) | 1.73 (1.24-2.43) |
| Nursing home | 1 (0.0) | 3 (0.1) | 0.18 (0.09-1.00) | 0.07 (0.03-1.00) | 5 (0.1) | 0 (0) | 1.00 (1.00-1.00) | 1.00 (1.00-1.00) | 0 | 0 |  |  |
| **Living with a child** |  |  |  |  |  |  |  |  |  |  |  |  |
| Attending daycare | 420 (3.7) | 90 (3.2) | 1.17 (1.07-1.28) | 1.3 (1.15-1.47) | 167 (3.5) | 30 (2.5) | 1.41 (1.23-1.61) | 1.61 (1.28-2.06) | 297 (2.6) | 55 (2.0) | 1.36 (1.22-1.49) | 1.45 (1.31-1.68) |
| Attended for by a professional in-home caregiver | 291 (2.6) | 66 (2.3) | 1.11 (0.99-1.21) | 1.44 (1.25-1.60) | 103 (2.1) | 32 (2.7) | 0.79 (0.69-0.91) | 0.82 (0.67-1.01) | 292 (2.6) | 54 (1.9) | 1.36 (1.23-1.50) | 1.25 (1.08-1.44) |
| Attending reschool | 938 (8.3) | 227 (8.1) | 1.03 (0.99-1.09) | 1.13 (1.05-1.20) | 610 (12.7) | 91 (7.6) | 1.78 (1.65-1.86) | 1.58 (1.40-1.71) | 1118 (9.9) | 218 (7.8) | 1.31 (1.26-1.37) | 1.25 (1.13-1.34) |
| Attending primary school | 1640 (14.6) | 451 (16.0) | 0.89 (0.86-0.93) | 1.00 (0.95-1.06) | 1044 (21.7) | 197 (16.3) | 1.41 (1.34-1.49) | 1.32 (1.19-1.45) | 2296 (20.4) | 389 (13.9) | 1.60 (1.55-1.65) | 1.61 (1.50-1.71) |
| Attending middle school | 1725 (15.3) | 422 (15.0) | 1.02 (0.99-1.07) | 1.13 (1.06-1.21) | 847 (17.6) | 164 (13.6) | 1.35 (1.29-1.42) | 1.52 (1.40-1.68) | 1786 (15.9) | 391 (13.9) | 1.17 (1.12-1.21) | 1.17 (1.07-1.22) |
| Attending high school | 1432 (12.7) | 381 (13.5) | 0.93 (0.89-0.97) | 1.05 (0.99-1.13) | 611 (12.7) | 163 (13.5) | 0.92 (0.87-0.98) | 0.88 (0.81-1.00) | 1284 (11.4) | 355 (12.6) | 0.89 (0.86-0.93) | 0.94 (0.88-1.00) |
| Attending university | 1075 (9.5) | 324 (11.5) | 0.82 (0.77-0.85) | 0.97 (0.90-1.06) | 451 (9.4) | 139 (11.5) | 0.79 (0.74-0.85) | 0.94 (0.81-1.05) | 881 (7.8) | 258 (9.2) | 0.84 (0.80-0.89) | 0.91 (0.84-0.97) |
| **Number of people in the household** |  |  |  |  |  |  |  |  |  |  |  |  |
| 1 | 2924 (26) | 573 (20.3) | 1 (ref) | 1 (ref) | 1024 (21.2) | 251 (20.8) | 1 (ref) | 1 (ref) | 2177 (19.4) | 594 (21.1) | 1 (ref) | 1 (ref) |
| 2 | 3025 (26.9) | 774 (27.5) | 0.77 (0.74-0.80) | 0.77 (0.74-0.82) | 1225 (25.4) | 346 (28.7) | 0.87 (0.81-0.93) | 0.89 (0.82-0.98) | 3667 (32.6) | 967 (34.4) | 1.04 (0.99-1.08) | 1.07 (1.02-1.15) |
| 3 | 2094 (18.6) | 625 (22.2) | 0.66 (0.62-0.69) | 0.68 (0.63-0.72) | 907 (18.8) | 263 (21.8) | 0.85 (0.78-0.91) | 0.90 (0.81-1.01) | 2079 (18.5) | 546 (19.4) | 1.04 (0.99-1.09) | 1.05 (0.96-1.14) |
| 4 | 2137 (19) | 610 (21.7) | 0.69 (0.66-0.73) | 0.75 (0.69-0.82) | 1049 (21.8) | 242 (20.1) | 1.07 (1.00-1.12) | 0.97 (0.87-1.09) | 2309 (20.5) | 517 (18.4) | 1.21 (1.15-1.29) | 1.13 (1.02-1.25) |
| 5 | 771 (6.8) | 169 (6) | 0.89 (0.84-0.96) | 0.99 (0.86-1.09) | 417 (8.7) | 72 (6.0) | 1.41 (1.30-1.55) | 1.25 (1.02-1.49) | 759 (6.7) | 154 (5.5) | 1.35 (1.26-1.42) | 1.17 (1.01-1.33) |
| 6+ | 313 (2.8) | 65 (2.3) | 0.94 (0.84-1.02) | 1.01 (0.83-1.14) | 198 (4.1) | 31 (2.6) | 1.57 (1.39-1.75) | 1.52 (1.22-1.74) | 257 (2.3) | 34 (1.2) | 2.06 (1.86-2.29) | 2.19 (1.84-2.58) |
| **Workplace** |  |  |  |  |  |  |  |  |  |  |  |  |
| No remote office, no open space | 1271 (11.3) | 340 (12.1) | 1 (ref) | 1 (ref) | 529 (11.0) | 148 (12.3) | 1 (ref) | 1 (ref) | 1418 (12.6) | 371 (13.2) | 1 (ref) | 1 (ref) |
| No remote office, open space | 895 (7.9) | 166 (5.9) | 1.45 (1.35-1.54) | 1.57 (1.41-1.74) | 354 (7.3) | 79 (6.6) | 1.25 (1.13-1.36) | 1.47 (1.33-1.64) | 879 (7.8) | 192 (6.8) | 1.20 (1.13-1.29) | 1.26 (1.16-1.37) |
| Not working | 2187 (19.4) | 521 (18.5) | 1.12 (1.06-1.20) | 1.59 (1.46-1.76) | 1129 (23.4) | 272 (22.6) | 1.17 (1.08-1.26) | 1.86 (1.61-2.06) | 2669 (23.7) | 759 (27.0) | 0.92 (0.88-0.98) | 1.52 (1.37-1.70) |
| Working in a non-office setting | 3848 (34.2) | 978 (34.7) | 1.05 (1.00-1.11) | 1.07 (1.00-1.15) | 1785 (37.0) | 420 (34.9) | 1.19 (1.09-1.27) | 1.19 (1.08-1.31) | 3438 (30.6) | 852 (30.3) | 1.06 (1.01-1.11) | 1.18 (1.10-1.26) |
| Partial remote office, no open space | 772 (6.9) | 293 (10.4) | 0.70 (0.66-0.75) | 0.76 (0.69-0.83) | 273 (5.7) | 108 (9.0) | 0.71 (0.63-0.79) | 0.72 (0.61-0.81) | 992 (8.8) | 258 (9.2) | 1.01 (0.94-1.07) | 0.77 (0.70-0.84) |
| Partial remote office, open space | 1126 (10) | 291 (10.3) | 1.04 (0.97-1.10) | 1.01 (0.91-1.12) | 343 (7.1) | 113 (9.4) | 0.85 (0.77-0.93) | 0.85 (0.72-0.98) | 1188 (10.6) | 240 (8.5) | 1.30 (1.22-1.38) | 1.09 (1.01-1.19) |
| Complete remote office | 1164 (10.3) | 227 (8.1) | 1.37 (1.29-1.47) | 1.15 (1.05-1.27) | 408 (8.5) | 65 (5.4) | 1.76 (1.60-1.92) | 1.64 (1.45-1.88) | 662 (5.9) | 140 (5.0) | 1.23 (1.12-1.37) | 1.01 (0.91-1.14) |
| **Gatherings** |  |  |  |  |  |  |  |  |  |  |  |  |
| Professional meeting | 2524 (22.4) | 703 (25.0) | 0.87 (0.84-0.90) | 0.88 (0.84-0.93) | 1037 (21.5) | 317 (26.3) | 0.77 (0.73-0.81) | 0.94 (0.86-1.00) | 4154 (36.9) | 860 (30.6) | 1.33 (1.28-1.38) | 1.25 (1.19-1.32) |
| Private meeting | 6192 (55.0) | 1704 (60.5) | 0.80 (0.78-0.82) | 0.78 (0.75-0.82) | 2386 (49.5) | 763 (63.3) | 0.57 (0.55-0.60) | 0.66 (0.62-0.70) | 6460 (57.4) | 1677 (59.6) | 0.91 (0.88-0.95) | 0.87 (0.83-0.91) |
| Religious gathering | 322 (2.9) | 119 (4.2) | 0.67 (0.62-0.71) | 0.90 (0.81-0.98) | 170 (3.5) | 59 (4.9) | 0.71 (0.64-0.79) | 0.74 (0.64-0.83) | 514 (4.6) | 125 (4.4) | 1.03 (0.94-1.11) | 1.16 (1.02-1.27) |
| **Retail, shops** |  |  |  |  |  |  |  |  |  |  |  |  |
| Mall | 2383 (21.2) | 833 (29.6) | 0.64 (0.62-0.66) | 0.77 (0.73-0.81) | 890 (18.5) | 346 (28.7) | 0.56 (0.53-0.59) | 0.73 (0.67-0.79) | 2456 (21.8) | 837 (29.8) | 0.66 (0.63-0.68) | 0.72 (0.68-0.74) |
| Market | 1574 (14.0) | 503 (17.9) | 0.75 (0.71-0.78) | 0.91 (0.85-0.97) | 696 (14.4) | 220 (18.3) | 0.76 (0.71-0.80) | 0.91 (0.82-0.98) | 1717 (15.3) | 494 (17.6) | 0.85 (0.81-0.89) | 0.90 (0.84-0.96) |
| Convenience store | 4226 (37.5) | 1069 (38) | 0.98 (0.95-1.01) | 1.23 (1.17-1.29) | 1714 (35.6) | 461 (38.3) | 0.89 (0.86-0.93) | 1.25 (1.16-1.34) | 4665 (41.5) | 1045 (37.2) | 1.20 (1.17-1.24) | 1.44 (1.37-1.51) |
| Supermarket | 5125 (45.5) | 1783 (63.3) | 0.48 (0.47-0.50) | 0.52 (0.50-0.53) | 2201 (45.7) | 780 (64.7) | 0.46 (0.44-0.48) | 0.54 (0.51-0.58) | 5890 (52.4) | 1861 (66.2) | 0.56 (0.54-0.58) | 0.58 (0.55-0.60) |
| Other shop | 310 (2.8) | 144 (5.1) | 0.52 (0.48-0.56) | 0.51 (0.46-0.56) | 156 (3.2) | 52 (4.3) | 0.74 (0.66-0.84) | 0.72 (0.59-0.85) | 404 (3.6) | 151 (5.4) | 0.66 (0.60-0.71) | 0.61 (0.55-0.68) |
| Beauty salon | 938 (8.3) | 240 (8.5) | 0.98 (0.93-1.02) | 1.12 (1.04-1.18) | 328 (6.8) | 110 (9.1) | 0.72 (0.66-0.79) | 0.84 (0.76-0.96) | 512 (4.6) | 202 (7.2) | 0.62 (0.57-0.66) | 0.69 (0.62-0.75) |
| Hairdresser | 992 (8.8) | 416 (14.8) | 0.56 (0.52-0.58) | 0.61 (0.57-0.65) | 485 (10.1) | 221 (18.3) | 0.50 (0.47-0.53) | 0.56 (0.50-0.60) | 1107 (9.8) | 473 (16.8) | 0.54 (0.51-0.56) | 0.61 (0.57-0.65) |
| Take-away delivery | 4353 (38.6) | 1132 (40.2) | 0.94 (0.91-0.97) | 1.07 (1.03-1.12) | 1743 (36.2) | 445 (36.9) | 0.97 (0.93-1.01) | 1.19 (1.14-1.26) | 3848 (34.2) | 942 (33.5) | 1.03 (1.00-1.07) | 1.13 (1.08-1.21) |
| Home delivery | 2881 (25.6) | 694 (24.6) | 1.05 (1.02-1.09) | 1.01 (0.96-1.06) | 1093 (22.7) | 290 (24.1) | 0.93 (0.87-0.97) | 0.99 (0.91-1.07) | 2205 (19.6) | 605 (21.5) | 0.89 (0.86-0.92) | 0.82 (0.78-0.87) |
| **Shared transport** |  |  |  |  |  |  |  |  |  |  |  |  |
| Metro | 2207 (19.6) | 587 (20.8) | 0.92 (0.89-0.96) | 1.02 (0.95-1.08) | 756 (15.7) | 227 (18.8) | 0.81 (0.74-0.85) | 1.07 (0.98-1.17) | 2091 (18.6) | 517 (18.4) | 1.01 (0.97-1.05) | 1.07 (1.00-1.14) |
| Tram | 923 (8.2) | 356 (12.6) | 0.61 (0.59-0.65) | 0.72 (0.67-0.77) | 336 (7.0) | 138 (11.5) | 0.58 (0.53-0.63) | 0.71 (0.64-0.81) | 779 (6.9) | 289 (10.3) | 0.65 (0.61-0.69) | 0.68 (0.62-0.75) |
| Short-distance bus | 1841 (16.3) | 632 (22.4) | 0.67 (0.65-0.70) | 0.75 (0.71-0.78) | 666 (13.8) | 259 (21.5) | 0.59 (0.55-0.62) | 0.58 (0.53-0.63) | 1388 (12.3) | 533 (19.0) | 0.60 (0.58-0.63) | 0.61 (0.57-0.65) |
| Short-distance train | 1072 (9.5) | 392 (13.9) | 0.65 (0.62-0.68) | 0.60 (0.55-0.64) | 281 (5.8) | 86 (7.1) | 0.81 (0.72-0.89) | 0.98 (0.81-1.10) | 859 (7.6) | 196 (7.0) | 1.11 (1.04-1.16) | 0.94 (0.86-1.02) |
| Airplane travel | 698 (6.2) | 96 (3.4) | 1.87 (1.75-1.99) | 1.67 (1.52-1.88) | 325 (6.7) | 62 (5.1) | 1.33 (1.23-1.45) | 1.20 (1.03-1.41) | 493 (4.4) | 85 (3.0) | 1.47 (1.37-1.56) | 1.44 (1.29-1.64) |
| Boat travel | 77 (0.7) | 18 (0.6) | 1.07 (0.86-1.22) | 0.90 (0.65-1.14) | 58 (1.2) | 10 (0.8) | 1.43 (1.20-1.74) | 1.80 (1.38-2.22) | 29 (0.3) | 9 (0.3) | 0.81 (0.58-1.00) | 0.61 (0.42-0.97) |
| Long-distance bus travel | 214 (1.9) | 71 (2.5) | 0.75 (0.67-0.82) | 0.72 (0.61-0.80) | 86 (1.8) | 27 (2.2) | 0.79 (0.69-0.91) | 1.18 (0.94-1.48) | 155 (1.4) | 46 (1.6) | 0.85 (0.74-0.96) | 0.88 (0.74-1.05) |
| Long-distance train travel | 950 (8.4) | 217 (7.7) | 1.10 (1.04-1.16) | 1.36 (1.25-1.46) | 295 (6.1) | 84 (7.0) | 0.87 (0.80-0.95) | 1.32 (1.12-1.50) | 856 (7.6) | 142 (5.0) | 1.55 (1.45-1.67) | 1.80 (1.63-1.94) |
| Car-pooling with relatives | 2996 (26.6) | 669 (23.8) | 1.16 (1.13-1.02) | 1.27 (1.21-1.32) | 1080 (22.4) | 295 (24.5) | 0.89 (0.84-0.95) | 1.19 (1.07-1.29) | 2914 (25.9) | 561 (20.0) | 1.40 (1.36-1.45) | 1.68 (1.62-1.77) |
| Car-pooling booked through platform | 180 (1.6) | 80 (2.8) | 0.55 (0.49-0.63) | 0.48 (0.41-0.55) | 58 (1.2) | 30 (2.5) | 0.48 (0.39-0.57) | 0.48 (0.39-0.60) | 115 (1.0) | 58 (2.1) | 0.49 (0.42-0.56) | 0.45 (0.36-0.52) |
| Taxi | 1110 (9.9) | 162 (5.8) | 1.79 (1.71-1.88) | 1.54 (1.42-1.69) | 367 (7.6) | 60 (5.0) | 1.57 (1.44-1.72) | 1.89 (1.68-2.25) | 845 (7.5) | 156 (5.5) | 1.38 (1.32-1.48) | 1.22 (1.10-1.33) |
| **Leisure activities** |  |  |  |  |  |  |  |  |  |  |  |  |
| Any cultural venue |  |  |  |  |  |  |  |  |  |  |  |  |
| Movie theatre | 841 (7.5) | 365 (13.0) | 0.54 (0.51-0.57) | 0.66 (0.61-0.72) | 285 (5.9) | 131 (10.9) | 0.52 (0.47-0.57) | 0.66 (0.57-0.74) | 1069 (9.5) | 368 (13.1) | 0.70 (0.66-0.73) | 0.71 (0.65-0.75) |
| Concert | 207 (1.8) | 46 (1.6) | 1.13 (0.99-1.23) | 0.85 (0.70-0.94) | 51 (1.1) | 15 (1.2) | 0.85 (0.66-1.05) | 1.51 (1.18-2.03) | 519 (4.6) | 89 (3.2) | 1.48 (1.36-1.60) | 1.31 (1.21-1.44) |
| Museum | 246 (2.2) | 124 (4.4) | 0.48 (0.44-0.53) | 0.59 (0.52-0.67) | 109 (2.3) | 61 (5.1) | 0.43 (0.36-0.50) | 0.61 (0.48-0.72) | 381 (3.4) | 105 (3.7) | 0.91 (0.82-0.97) | 0.87 (0.76-0.97) |
| Theatre | 134 (1.2) | 49 (1.7) | 0.68 (0.60-0.75) | 0.85 (0.73-0.95) | 47 (1.0) | 15 (1.2) | 0.76 (0.61-0.97) | 1.03 (0.75-1.46) | 451 (4.0) | 86 (3.1) | 1.33 (1.21-1.43) | 1.45 (1.26-1.63) |
| Indoor sports practice | 781 (6.9) | 193 (6.9) | 1.01 (0.95-1.08) | 1.08 (0.98-1.16) | 335 (7.0) | 91 (7.6) | 0.91 (0.84-0.99) | 1.20 (1.01-1.36) | 1233 (11) | 296 (10.5) | 1.05 (1.00-1.10) | 1.05 (0.98-1.11) |
| Swimming pool | 650 (5.8) | 242 (8.6) | 0.65 (0.61-0.69) | 0.76 (0.69-0.83) | 296 (6.1) | 99 (8.2) | 0.73 (0.67-0.79) | 0.84 (0.73-0.95) | 628 (5.6) | 190 (6.8) | 0.81 (0.76-0.88) | 0.84 (0.76-0.92) |
| Indoor martial arts facility | 34 (0.3) | 27 (1.0) | 0.31 (0.24-0.43) | 0.42 (0.33-0.61) | 19 (0.4) | 17 (1.4) | 0.29 (0.17-0.36) | 0.44 (0.29-0.66) | 159 (1.4) | 52 (1.8) | 0.76 (0.67-0.85) | 0.85 (0.73-0.98) |
| Indoor gymnastics facility | 119 (1.1) | 41 (1.5) | 0.72 (0.63-0.82) | 0.92 (0.79-1.09) | 93 (1.9) | 32 (2.7) | 0.72 (0.63-0.82) | 0.71 (0.57-0.85) | 577 (5.1) | 166 (5.9) | 0.86 (0.80-0.92) | 0.84 (0.76-0.90) |
| Outdoor sports practice | 2763 (24.5) | 865 (30.7) | 0.73 (0.70-0.75) | 0.75 (0.72-0.78) | 1183 (24.5) | 363 (30.1) | 0.75 (0.73-0.79) | 0.78 (0.73-0.83) | 2621 (23.3) | 792 (28.2) | 0.77 (0.75-0.80) | 0.68 (0.65-0.71) |
| Bar | 3480 (30.9) | 650 (23.1) | 1.49 (1.44-1.53) | 1.57 (1.50-1.64) | 907 (18.8) | 259 (21.5) | 0.85 (0.80-0.89) | 1.28 (1.14-1.39) | 1931 (17.2) | 479 (17.0) | 1.01 (0.97-1.04) | 1.04 (0.98-1.10) |
| Restaurant | 5311 (47.1) | 1408 (50.0) | 0.89 (0.86-0.92) | 0.95 (0.89-0.99) | 1830 (38.0) | 580 (48.1) | 0.66 (0.63-0.69) | 0.93 (0.86-0.99) | 4463 (39.7) | 1188 (42.2) | 0.90 (0.87-0.93) | 0.88 (0.84-0.92) |
| Party | 1888 (16.8) | 216 (7.7) | 2.42 (2.33-2.50) | 2.66 (2.52-2.84) |  |  |  |  |  |  |  |  |
| Night-club |  |  |  |  | 302 (6.3) | 44 (3.7) | 1.77 (1.62-1.93) | 2.34 (1.93-2.61) | 551 (4.9) | 72 (2.6) | 1.96 (1.83-2.10) | 2.95 (2.64-3.28) |
| Private party |  |  |  |  | 427 (8.9) | 190 (15.8) | 0.52 (0.48-0.56) | 0.65 (0.57-0.71) | 1658 (14.7) | 482 (17.1) | 0.84 (0.79-0.88) | 0.82 (0.76-0.87) |

Legend: Adjusted odds ratios estimated in models adjusted for all variables shown in the table, as well as week of exposure, health status (diabetes, hypertension, respiratory disease, coronary artery disease, immunosuppressive treatment, body-mass index categorized as <18.5 kg/m², [18.5-25[, [25-30[, and ≥30 kg/m²), COVID-19 vaccine status (categorized as number of doses and time since last dose as <90 days, 90-179 days, ≥180 days, with a specific category for participants with missing data for the date of the last dose), past SARS-CoV-2 infection (categorized as 61-180 days prior or over 180 days prior), smoking status, level of education, and professional category (of the person in the household with the highest income for the first two periods, of the participant for the following periods). Empty cells reflect changes of the questionnaire or periods when specific settings were closed.

**Table S3: Exposure prevalence, odds ratios of SARS-CoV-2 infection in univariable and multivariable estimates in a case-control study conducted in mainland France (2020-2022) – Results for the last three periods of the study (December 20, 2021 to October 2, 2022)**

| Period (onset date) | 7: 12/20/2021 | | | | 8: 03/18/2022 | | | | 9: 05/20/2022 | | | |
| --- | --- | --- | --- | --- | --- | --- | --- | --- | --- | --- | --- | --- |
|  | Cases | Controls | OR (95% CI) | Adjusted OR (95% CI) | Cases | Controls | OR (95% CI) | Adjusted OR (95% CI) | Cases | Controls | OR (95% CI) | Adjusted OR (95% CI) |
| Male sex | 16356 (37.1) | 4089 (37.1) | 1.00 (1.00-1.00) | 1.12 (1.11-1.14) | 16200 (40.9) | 4050 (40.9) | 1.00 (1.00-1.00) | 1.10 (1.09-1.12) | 8148 (28.5) | 2037 (28.5) | 1.00 (1.00-1.00) | 1.16 (1.14-1.19) |
| **Age (years)** |  |  |  |  |  |  |  |  |  |  |  |  |
| 18-29 | 3640 (8.2) | 910 (8.2) | 1 (ref) | 1 (ref) | 3152 (7.9) | 788 (7.9) | 1 (ref) | 1 (ref) | 1952 (6.8) | 488 (6.8) | 1 (ref) | 1 (ref) |
| 30-39 | 7776 (17.6) | 1944 (17.6) | 1.00 (1.00-1.00) | 0.67 (0.66-0.69) | 7124 (18.0) | 1781 (18.0) | 1.00 (1.00-1.00) | 0.72 (0.69-0.75) | 5692 (19.9) | 1423 (19.9) | 1.00 (1.00-1.00) | 0.83 (0.79-0.87) |
| 40-49 | 10628 (24.1) | 2657 (24.1) | 1.00 (1.00-1.00) | 0.72 (0.70-0.74) | 9500 (24.0) | 2375 (24.0) | 1.00 (1.00-1.00) | 0.68 (0.65-0.70) | 7300 (25.5) | 1825 (25.5) | 1.00 (1.00-1.00) | 0.86 (0.83-0.91) |
| 50-59 | 9616 (21.8) | 2404 (21.8) | 1.00 (1.00-1.00) | 0.97 (0.94-1.00) | 9204 (23.2) | 2301 (23.2) | 1.00 (1.00-1.00) | 0.73 (0.71-0.76) | 6668 (23.3) | 1667 (23.3) | 1.00 (1.00-1.00) | 0.81 (0.77-0.85) |
| 60-69 | 8588 (19.5) | 2147 (19.5) | 1.00 (1.00-1.00) | 1.29 (1.22-1.35) | 6900 (17.4) | 1725 (17.4) | 1.00 (1.00-1.00) | 0.92 (0.88-0.97) | 4864 (17.0) | 1216 (17.0) | 1.00 (1.00-1.00) | 0.95 (0.89-1.00) |
| 70+ | 3888 (8.8) | 972 (8.8) | 1.00 (1.00-1.00) | 1.24 (1.17-1.31) | 3772 (9.5) | 943 (9.5) | 1.00 (1.00-1.00) | 0.96 (0.90-1.02) | 2140 (7.5) | 535 (7.5) | 1.00 (1.00-1.00) | 0.92 (0.85-0.98) |
| **Population in the area of residence** |  |  |  |  |  |  |  |  |  |  |  |  |
| <5000 inhabitants | 12564 (28.5) | 3141 (28.5) | 1 (ref) | 1 (ref) | 11072 (27.9) | 2768 (27.9) | 1 (ref) | 1 (ref) | 7092 (24.8) | 1773 (24.8) | 1 (ref) | 1 (ref) |
| 5000 – 19,999 inhabitants | 4160 (9.4) | 1040 (9.4) | 1.00 (1.00-1.00) | 1.02 (1.00-1.04) | 4100 (10.3) | 1025 (10.3) | 1.00 (1.00-1.00) | 0.99 (0.98-1.01) | 1796 (6.3) | 449 (6.3) | 1.00 (1.00-1.00) | 1.01 (0.98-1.04) |
| 20,000 – 99,999 inhabitants | 4888 (11.1) | 1222 (11.1) | 1.00 (1.00-1.00) | 1.09 (1.06-1.10) | 4888 (12.3) | 1222 (12.3) | 1.00 (1.00-1.00) | 1.04 (1.01-1.06) | 2648 (9.3) | 662 (9.3) | 1.00 (1.00-1.00) | 1.07 (1.04-1.09) |
| Over 100,000 inhabitants | 15140 (34.3) | 3785 (34.3) | 1.00 (1.00-1.00) | 1.00 (0.99-1.02) | 13112 (33.1) | 3278 (33.1) | 1.00 (1.00-1.00) | 0.95 (0.93-0.97) | 10212 (35.7) | 2553 (35.7) | 1.00 (1.00-1.00) | 0.97 (0.95-0.99) |
| Greater Paris area | 7384 (16.7) | 1846 (16.7) | 1.00 (1.00-1.00) | 0.91 (0.87-0.95) | 6480 (16.3) | 1620 (16.3) | 1.00 (1.00-1.00) | 0.78 (0.75-0.82) | 6868 (24.0) | 1717 (24.0) | 1.00 (1.00-1.00) | 0.86 (0.8-0.92) |
| **Region of residence** |  |  |  |  |  |  |  |  |  |  |  |  |
| Ile-de-France | 8012 (18.2) | 2003 (18.2) | 1 (ref) | 1 (ref) | 7252 (18.3) | 1813 (18.3) | 1 (ref) | 1 (ref) | 7144 (25.0) | 1786 (25.0) | 1 (ref) | 1 (ref) |
| Auverge-Rhône-Alpes | 5308 (12.0) | 1327 (12.0) | 1.00 (1.00-1.00) | 1.04 (1.00-1.09) | 4916 (12.4) | 1229 (12.4) | 1.00 (1.00-1.00) | 1.01 (0.96-1.05) | 3844 (13.4) | 961 (13.4) | 1.00 (1.00-1.00) | 0.99 (0.92-1.05) |
| Occitanie | 4968 (11.3) | 1242 (11.3) | 1.00 (1.00-1.00) | 1.04 (1.00-1.08) | 4012 (10.1) | 1003 (10.1) | 1.00 (1.00-1.00) | 1.02 (0.98-1.06) | 3128 (10.9) | 782 (10.9) | 1.00 (1.00-1.00) | 0.99 (0.93-1.05) |
| Provence-Alpes-Côte d'Azur and Corsica | 3336 (7.6) | 834 (7.6) | 1.00 (1.00-1.00) | 1.16 (1.11-1.21) | 3208 (8.1) | 802 (8.1) | 1.00 (1.00-1.00) | 1.10 (1.06-1.15) | 2576 (9.0) | 644 (9.0) | 1.00 (1.00-1.00) | 1.10 (1.03-1.20) |
| Grand Est | 4012 (9.1) | 1003 (9.1) | 1.00 (1.00-1.00) | 1.08 (1.04-1.13) | 3596 (9.1) | 899 (9.1) | 1.00 (1.00-1.00) | 1.01 (0.96-1.04) | 2356 (8.2) | 589 (8.2) | 1.00 (1.00-1.00) | 0.95 (0.9-1.02) |
| Nouvelle-Aquitaine | 4640 (10.5) | 1160 (10.5) | 1.00 (1.00-1.00) | 1.09 (1.04-1.13) | 3360 (8.5) | 840 (8.5) | 1.00 (1.00-1.00) | 1.01 (0.96-1.06) | 2624 (9.2) | 656 (9.2) | 1.00 (1.00-1.00) | 1.00 (0.92-1.06) |
| Hauts-de-France | 3912 (8.9) | 978 (8.9) | 1.00 (1.00-1.00) | 1.13 (1.08-1.18) | 3764 (9.5) | 941 (9.5) | 1.00 (1.00-1.00) | 1.02 (0.98-1.07) | 2444 (8.5) | 611 (8.5) | 1.00 (1.00-1.00) | 0.99 (0.93-1.05) |
| Pays de la Loire | 2396 (5.4) | 599 (5.4) | 1.00 (1.00-1.00) | 1.05 (1.00-1.10) | 2468 (6.2) | 617 (6.2) | 1.00 (1.00-1.00) | 1.02 (0.97-1.07) | 1188 (4.2) | 297 (4.2) | 1.00 (1.00-1.00) | 1.01 (0.92-1.07) |
| Bretagne | 2800 (6.3) | 700 (6.3) | 1.00 (1.00-1.00) | 0.98 (0.95-1.03) | 2752 (6.9) | 688 (6.9) | 1.00 (1.00-1.00) | 0.94 (0.89-0.97) | 1204 (4.2) | 301 (4.2) | 1.00 (1.00-1.00) | 1.02 (0.94-1.10) |
| Normandie | 1716 (3.9) | 429 (3.9) | 1.00 (1.00-1.00) | 1.08 (1.04-1.14) | 1612 (4.1) | 403 (4.1) | 1.00 (1.00-1.00) | 1.04 (0.99-1.10) | 840 (2.9) | 210 (2.9) | 1.00 (1.00-1.00) | 0.96 (0.88-1.02) |
| Bourgogne-Franche-Comté | 1760 (4.0) | 440 (4.0) | 1.00 (1.00-1.00) | 1.02 (0.98-1.06) | 1260 (3.2) | 315 (3.2) | 1.00 (1.00-1.00) | 1.00 (0.96-1.05) | 624 (2.2) | 156 (2.2) | 1.00 (1.00-1.00) | 0.93 (0.87-1.00) |
| Centre-Val de Loire | 1276 (2.9) | 319 (2.9) | 1.00 (1.00-1.00) | 1.00 (0.96-1.06) | 1452 (3.7) | 363 (3.7) | 1.00 (1.00-1.00) | 0.94 (0.90-0.99) | 644 (2.3) | 161 (2.3) | 1.00 (1.00-1.00) | 0.97 (0.90-1.05) |
| **Education level** |  |  |  |  |  |  |  |  |  |  |  |  |
| No diploma | 943 (2.1) | 233 (2.1) | 1 (ref) | 1 (ref) | 729 (1.8) | 186 (1.9) | 1 (ref) | 1 (ref) | 554 (1.9) | 129 (1.8) | 1 (ref) | 1 (ref) |
| Pre-high school diploma | 6975 (15.8) | 2231 (20.2) | 0.77 (0.73-0.82) | 0.67 (0.63-0.72) | 6158 (15.5) | 2141 (21.6) | 0.73 (0.69-0.78) | 0.60 (0.55-0.65) | 4139 (14.5) | 1288 (18) | 0.75 (0.70-0.80) | 0.61 (0.56-0.66) |
| High-school diploma | 8006 (18.1) | 2804 (25.4) | 0.71 (0.66-0.74) | 0.61 (0.57-0.65) | 7321 (18.5) | 2533 (25.6) | 0.73 (0.70-0.79) | 0.57 (0.53-0.63) | 5108 (17.8) | 1791 (25.0) | 0.67 (0.62-0.72) | 0.54 (0.49-0.59) |
| Bachelor's degree | 16438 (37.2) | 4080 (37.0) | 1.00 (0.94-1.05) | 0.74 (0.69-0.78) | 14819 (37.4) | 3665 (37) | 1.03 (0.97-1.1) | 0.70 (0.65-0.77) | 10773 (37.6) | 2788 (39.0) | 0.90 (0.84-0.97) | 0.65 (0.60-0.70) |
| Master's degree or higher | 11773 (26.7) | 1686 (15.3) | 1.73 (1.63-1.82) | 1.10 (1.03-1.18) | 10626 (26.8) | 1388 (14) | 1.95 (1.84-2.09) | 1.09 (1.01-1.20) | 8043 (28.1) | 1158 (16.2) | 1.62 (1.51-1.73) | 0.99 (0.90-1.08) |
| **Health conditions** |  |  |  |  |  |  |  |  |  |  |  |  |
| Diabetes mellitus | 1751 (4.0) | 640 (5.8) | 0.67 (0.65-0.70) | 0.69 (0.67-0.73) | 1660 (4.2) | 637 (6.4) | 0.64 (0.61-0.66) | 0.70 (0.67-0.74) | 1098 (3.8) | 414 (5.8) | 0.65 (0.61-0.69) | 0.68 (0.64-0.72) |
| Hypertension | 5907 (13.4) | 1677 (15.2) | 0.86 (0.84-0.88) | 0.90 (0.88-0.93) | 5662 (14.3) | 1586 (16) | 0.87 (0.85-0.90) | 0.92 (0.89-0.95) | 3696 (12.9) | 989 (13.8) | 0.92 (0.90-0.95) | 0.94 (0.90-0.97) |
| Chronic respiratory disease | 3632 (8.2) | 806 (7.3) | 1.14 (1.10-1.17) | 1.30 (1.25-1.34) | 3455 (8.7) | 705 (7.1) | 1.25 (1.21-1.28) | 1.40 (1.36-1.47) | 2547 (8.9) | 533 (7.5) | 1.21 (1.17-1.26) | 1.34 (1.26-1.40) |
| Coronary artery disease | 636 (1.4) | 152 (1.4) | 1.04 (0.98-1.13) | 1.16 (1.05-1.29) | 626 (1.6) | 111 (1.1) | 1.42 (1.33-1.50) | 1.59 (1.44-1.70) | 347 (1.2) | 68 (1.0) | 1.28 (1.18-1.39) | 1.46 (1.29-1.63) |
| **Body-mass index (kg/m²)** |  |  |  |  |  |  |  |  |  |  |  |  |
| Healthy weight (≥18.5 & <25) | 22002 (49.9) | 5060 (45.9) | 1 (ref) | 1 (ref) | 18968 (47.8) | 4322 (43.6) | 1 (ref) | 1 (ref) | 14030 (49.0) | 3387 (47.3) | 1 (ref) | 1 (ref) |
| Underweight (<18.5) | 1192 (2.7) | 448 (4.1) | 0.61 (0.58-0.64) | 0.60 (0.56-0.63) | 1003 (2.5) | 353 (3.6) | 0.65 (0.61-0.68) | 0.65 (0.60-0.70) | 912 (3.2) | 301 (4.2) | 0.73 (0.70-0.77) | 0.70 (0.66-0.74) |
| Overweight (≥25 & <30) | 13865 (31.4) | 3453 (31.3) | 0.92 (0.91-0.94) | 0.96 (0.94-0.98) | 12878 (32.5) | 3245 (32.7) | 0.90 (0.89-0.92) | 0.94 (0.91-0.96) | 8616 (30.1) | 2119 (29.6) | 0.98 (0.96-1.00) | 1.02 (1.00-1.05) |
| Obesity (≥30) | 7077 (16.0) | 2073 (18.8) | 0.79 (0.77-0.80) | 0.86 (0.83-0.88) | 6803 (17.2) | 1993 (20.1) | 0.78 (0.76-0.79) | 0.82 (0.79-0.84) | 5058 (17.7) | 1347 (18.8) | 0.91 (0.89-0.93) | 0.99 (0.96-1.03) |
| **Housing** |  |  |  |  |  |  |  |  |  |  |  |  |
| Individual house | 29262 (66.3) | 6904 (62.6) | 1 (ref) | 1 (ref) | 25967 (65.5) | 6272 (63.3) | 1 (ref) | 1 (ref) | 17120 (59.8) | 4192 (58.6) | 1 (ref) | 1 (ref) |
| Apartment | 14757 (33.4) | 4101 (37.2) | 0.85 (0.84-0.86) | 0.96 (0.94-0.99) | 13593 (34.3) | 3608 (36.4) | 0.91 (0.90-0.92) | 0.99 (0.96-1.02) | 11436 (40.0) | 2942 (41.1) | 0.95 (0.94-0.97) | 0.95 (0.92-0.98) |
| Shelter | 97 (0.2) | 23 (0.2) | 0.99 (0.85-1.18) | 1.17 (0.98-1.52) | 79 (0.2) | 26 (0.3) | 0.75 (0.58-0.88) | 1.00 (0.66-1.38) | 51 (0.2) | 15 (0.2) | 0.83 (0.67-1.05) | 1.07 (0.86-1.42) |
| Nursing home | 20 (0.0) | 7 (0.1) | 0.67 (0.50-0.81) | 0.84 (0.55-1.23) | 13 (0) | 7 (0.1) | 0.45 (0.24-0.73) | 0.46 (0.22-0.76) | 9 (0.0) | 5 (0.1) | 0.44 (0.20-0.69) | 2.07 (0.49-6.51) |
| **Living with a child** |  |  |  |  |  |  |  |  |  |  |  |  |
| Attending daycare | 1219 (2.8) | 183 (1.7) | 1.68 (1.62-1.75) | 1.58 (1.46-1.68) | 964 (2.4) | 183 (1.8) | 1.33 (1.26-1.4) | 1.29 (1.18-1.40) | 768 (2.7) | 180 (2.5) | 1.07 (1.02-1.12) | 1.25 (1.13-1.34) |
| Attended for by a professional in-home caregiver | 1499 (3.4) | 214 (1.9) | 1.78 (1.69-1.85) | 1.53 (1.45-1.62) | 1263 (3.2) | 206 (2.1) | 1.55 (1.49-1.62) | 1.48 (1.38-1.58) | 915 (3.2) | 138 (1.9) | 1.68 (1.58-1.76) | 1.63 (1.53-1.76) |
| Attending reschool | 4446 (10.1) | 759 (6.9) | 1.51 (1.49-1.55) | 1.56 (1.51-1.64) | 3115 (7.9) | 695 (7.0) | 1.13 (1.10-1.16) | 1.22 (1.15-1.28) | 2362 (8.3) | 583 (8.1) | 1.01 (0.99-1.04) | 1.20 (1.12-1.26) |
| Attending primary school | 7667 (17.4) | 1397 (12.7) | 1.45 (1.42-1.48) | 1.52 (1.46-1.57) | 5864 (14.8) | 1304 (13.2) | 1.15 (1.12-1.17) | 1.43 (1.36-1.49) | 3849 (13.5) | 1035 (14.5) | 0.92 (0.90-0.94) | 1.20 (1.16-1.26) |
| Attending middle school | 6798 (15.4) | 1404 (12.7) | 1.25 (1.23-1.27) | 1.31 (1.27-1.34) | 5431 (13.7) | 1365 (13.8) | 0.99 (0.97-1.02) | 1.11 (1.07-1.16) | 3602 (12.6) | 1040 (14.5) | 0.85 (0.82-0.87) | 1.02 (0.97-1.06) |
| Attending high school | 5266 (11.9) | 1251 (11.3) | 1.06 (1.03-1.08) | 1.14 (1.10-1.18) | 4414 (11.1) | 1157 (11.7) | 0.95 (0.92-0.97) | 1.10 (1.04-1.14) | 3205 (11.2) | 881 (12.3) | 0.90 (0.88-0.93) | 1.09 (1.05-1.14) |
| Attending university | 3235 (7.3) | 898 (8.1) | 0.89 (0.87-0.92) | 0.85 (0.82-0.90) | 2918 (7.4) | 796 (8.0) | 0.91 (0.88-0.95) | 0.93 (0.89-0.97) | 2448 (8.6) | 668 (9.3) | 0.91 (0.88-0.94) | 0.93 (0.89-0.97) |
| **Number of people in the household** |  |  |  |  |  |  |  |  |  |  |  |  |
| 1 | 8216 (18.6) | 2412 (21.9) | 1 (ref) | 1 (ref) | 8055 (20.3) | 2048 (20.7) | 1 (ref) | 1 (ref) | 6380 (22.3) | 1438 (20.1) | 1 (ref) | 1 (ref) |
| 2 | 15949 (36.1) | 4179 (37.9) | 1.12 (1.10-1.14) | 1.05 (1.02-1.08) | 14900 (37.6) | 3702 (37.3) | 1.02 (1.00-1.05) | 0.98 (0.96-1.02) | 10069 (35.2) | 2473 (34.6) | 0.92 (0.89-0.94) | 0.93 (0.89-0.96) |
| 3 | 7888 (17.9) | 1927 (17.5) | 1.20 (1.17-1.23) | 1.05 (1.02-1.08) | 7195 (18.1) | 1807 (18.2) | 1.01 (0.98-1.04) | 0.91 (0.88-0.96) | 5409 (18.9) | 1398 (19.5) | 0.87 (0.85-0.89) | 0.81 (0.77-0.85) |
| 4 | 8586 (19.5) | 1787 (16.2) | 1.41 (1.38-1.45) | 1.03 (0.99-1.07) | 7024 (17.7) | 1635 (16.5) | 1.09 (1.07-1.12) | 0.90 (0.85-0.94) | 4949 (17.3) | 1286 (18) | 0.87 (0.84-0.89) | 0.74 (0.70-0.79) |
| 5 | 2683 (6.1) | 573 (5.2) | 1.37 (1.33-1.43) | 0.99 (0.94-1.06) | 1927 (4.9) | 553 (5.6) | 0.89 (0.85-0.93) | 0.77 (0.71-0.85) | 1389 (4.9) | 437 (6.1) | 0.72 (0.69-0.75) | 0.68 (0.63-0.73) |
| 6+ | 814 (1.8) | 156 (1.4) | 1.54 (1.45-1.62) | 1.29 (1.18-1.42) | 552 (1.4) | 168 (1.7) | 0.84 (0.76-0.89) | 0.86 (0.79-0.95) | 419 (1.5) | 122 (1.7) | 0.77 (0.73-0.83) | 0.85 (0.77-0.98) |
| **Workplace** |  |  |  |  |  |  |  |  |  |  |  |  |
| No remote office, no open space | 4843 (11.0) | 1221 (11.1) | 1 (ref) | 1 (ref) | 4981 (12.6) | 1043 (10.5) | 1 (ref) | 1 (ref) | 3117 (10.9) | 803 (11.2) | 1 (ref) | 1 (ref) |
| No remote office, open space | 2566 (5.8) | 625 (5.7) | 1.04 (0.99-1.07) | 1.19 (1.12-1.24) | 2951 (7.4) | 571 (5.8) | 1.08 (1.04-1.13) | 1.23 (1.17-1.31) | 2099 (7.3) | 427 (6.0) | 1.26 (1.22-1.33) | 1.33 (1.26-1.40) |
| Not working | 11808 (26.8) | 3901 (35.4) | 0.76 (0.75-0.78) | 0.85 (0.81-0.88) | 10005 (25.2) | 3465 (35) | 0.60 (0.59-0.62) | 0.58 (0.55-0.62) | 6516 (22.8) | 2233 (31.2) | 0.75 (0.73-0.77) | 0.84 (0.80-0.90) |
| Working in a non-office setting | 11771 (26.7) | 2694 (24.4) | 1.10 (1.07-1.13) | 1.31 (1.26-1.37) | 10054 (25.4) | 2532 (25.5) | 0.83 (0.81-0.86) | 1.19 (1.15-1.24) | 6134 (21.4) | 1464 (20.5) | 1.08 (1.04-1.11) | 1.35 (1.29-1.41) |
| Partial remote office, no open space | 3391 (7.7) | 786 (7.1) | 1.09 (1.05-1.13) | 0.90 (0.87-0.95) | 2879 (7.3) | 602 (6.1) | 1.00 (0.96-1.04) | 0.86 (0.81-0.91) | 1737 (6.1) | 431 (6.0) | 1.04 (0.98-1.08) | 0.86 (0.82-0.91) |
| Partial remote office, open space | 3186 (7.2) | 694 (6.3) | 1.16 (1.11-1.20) | 1.00 (0.94-1.04) | 3330 (8.4) | 598 (6.0) | 1.17 (1.12-1.21) | 0.98 (0.93-1.03) | 2171 (7.6) | 486 (6.8) | 1.15 (1.10-1.20) | 0.95 (0.91-1.01) |
| Complete remote office | 2316 (5.2) | 421 (3.8) | 1.39 (1.32-1.44) | 1.15 (1.09-1.20) | 1577 (4.0) | 286 (2.9) | 1.16 (1.09-1.23) | 1.00 (0.92-1.07) | 1072 (3.7) | 236 (3.3) | 1.17 (1.10-1.24) | 1 (0.92-1.09) |
| On leave during the period of interest | 4254 (9.6) | 691 (6.3) | 1.55 (1.5-1.60) | 1.82 (1.75-1.92) | 3876 (9.8) | 816 (8.2) | 0.99 (0.96-1.04) | 1.18 (1.13-1.25) | 5771 (20.2) | 1074 (15.0) | 1.39 (1.34-1.43) | 1.58 (1.53-1.67) |
| **Gatherings** |  |  |  |  |  |  |  |  |  |  |  |  |
| Professional meeting | 11548 (26.2) | 2401 (21.8) | 1.28 (1.25-1.29) | 1.14 (1.11-1.18) | 12304 (31) | 2419 (24.4) | 1.39 (1.37-1.42) | 1.11 (1.07-1.14) | 7217 (25.2) | 1463 (20.5) | 1.31 (1.29-1.35) | 1.31 (1.27-1.37) |
| Private meeting | 19067 (43.2) | 4933 (44.7) | 0.94 (0.92-0.96) | 0.93 (0.91-0.95) | 16213 (40.9) | 4610 (46.5) | 0.80 (0.78-0.81) | 0.82 (0.79-0.84) | 11985 (41.9) | 3386 (47.3) | 0.80 (0.79-0.81) | 0.82 (0.80-0.84) |
| Religious gathering | 1715 (3.9) | 423 (3.8) | 1.01 (0.98-1.05) | 1.05 (1.00-1.09) | 1638 (4.1) | 477 (4.8) | 0.85 (0.81-0.89) | 0.94 (0.89-0.99) | 1036 (3.6) | 327 (4.6) | 0.78 (0.74-0.82) | 0.96 (0.90-1.01) |
| **Retail, shops** |  |  |  |  |  |  |  |  |  |  |  |  |
| Mall | 6813 (15.4) | 2550 (23.1) | 0.61 (0.59-0.62) | 0.68 (0.66-0.69) | 5768 (14.5) | 2147 (21.7) | 0.62 (0.60-0.63) | 0.72 (0.69-0.74) | 5244 (18.3) | 1726 (24.1) | 0.70 (0.69-0.72) | 0.79 (0.77-0.81) |
| Market | 4557 (10.3) | 1359 (12.3) | 0.82 (0.80-0.84) | 0.85 (0.83-0.88) | 4010 (10.1) | 1218 (12.3) | 0.80 (0.78-0.82) | 0.88 (0.84-0.92) | 3831 (13.4) | 1060 (14.8) | 0.89 (0.87-0.91) | 0.97 (0.93-1.01) |
| Convenience store | 15459 (35.0) | 3826 (34.7) | 1.01 (1.00-1.03) | 1.18 (1.15-1.2) | 13589 (34.3) | 3424 (34.5) | 0.99 (0.97-1.00) | 1.15 (1.12-1.18) | 9844 (34.4) | 2403 (33.6) | 1.04 (1.02-1.06) | 1.17 (1.13-1.20) |
| Supermarket | 21721 (49.2) | 7000 (63.4) | 0.56 (0.55-0.57) | 0.59 (0.58-0.60) | 19430 (49.0) | 6383 (64.4) | 0.53 (0.52-0.54) | 0.57 (0.56-0.59) | 13834 (48.3) | 4351 (60.8) | 0.60 (0.59-0.61) | 0.66 (0.64-0.67) |
| Other shop | 1420 (3.2) | 540 (4.9) | 0.65 (0.62-0.67) | 0.66 (0.62-0.69) | 1339 (3.4) | 472 (4.8) | 0.70 (0.66-0.73) | 0.71 (0.68-0.74) | 1103 (3.9) | 295 (4.1) | 0.93 (0.89-0.97) | 0.88 (0.83-0.95) |
| Beauty salon | 1512 (3.4) | 593 (5.4) | 0.63 (0.59-0.66) | 0.77 (0.72-0.81) | 1320 (3.3) | 529 (5.3) | 0.61 (0.58-0.65) | 0.72 (0.67-0.77) | 1290 (4.5) | 491 (6.9) | 0.64 (0.61-0.67) | 0.80 (0.76-0.85) |
| Hairdresser | 3158 (7.2) | 1434 (13.0) | 0.52 (0.50-0.53) | 0.57 (0.55-0.59) | 2980 (7.5) | 1279 (12.9) | 0.55 (0.53-0.57) | 0.64 (0.62-0.66) | 2056 (7.2) | 953 (13.3) | 0.50 (0.48-0.52) | 0.56 (0.54-0.59) |
| Take-away delivery | 12548 (28.4) | 3078 (27.9) | 1.03 (1.01-1.04) | 1.11 (1.08-1.13) | 10864 (27.4) | 2781 (28.0) | 0.97 (0.95-0.99) | 1.12 (1.09-1.14) | 8109 (28.3) | 1966 (27.5) | 1.04 (1.02-1.07) | 1.28 (1.23-1.31) |
| Home delivery | 5388 (12.2) | 1468 (13.3) | 0.91 (0.89-0.93) | 0.91 (0.88-0.94) | 4623 (11.7) | 1322 (13.3) | 0.86 (0.83-0.88) | 0.85 (0.82-0.89) | 3221 (11.3) | 994 (13.9) | 0.79 (0.76-0.81) | 0.80 (0.78-0.85) |
| **Shared transport** |  |  |  |  |  |  |  |  |  |  |  |  |
| Metro | 5139 (11.6) | 1244 (11.3) | 1.04 (1.01-1.06) | 1.07 (1.03-1.11) | 5097 (12.9) | 1141 (11.5) | 1.13 (1.10-1.16) | 1.16 (1.12-1.20) | 4414 (15.4) | 1008 (14.1) | 1.11 (1.09-1.15) | 1.15 (1.1-1.2) |
| Tram | 2447 (5.5) | 776 (7.0) | 0.78 (0.75-0.80) | 0.87 (0.82-0.91) | 2253 (5.7) | 700 (7.1) | 0.79 (0.76-0.82) | 0.84 (0.80-0.89) | 1844 (6.4) | 589 (8.2) | 0.77 (0.74-0.79) | 0.84 (0.8-0.88) |
| Short-distance bus | 4204 (9.5) | 1415 (12.8) | 0.71 (0.70-0.74) | 0.80 (0.77-0.83) | 4094 (10.3) | 1299 (13.1) | 0.76 (0.74-0.79) | 0.79 (0.76-0.82) | 3910 (13.7) | 1116 (15.6) | 0.86 (0.84-0.88) | 0.85 (0.81-0.88) |
| Short-distance train | 1987 (4.5) | 521 (4.7) | 0.95 (0.91-0.99) | 0.91 (0.86-0.96) | 2179 (5.5) | 591 (6.0) | 0.92 (0.88-0.95) | 0.85 (0.80-0.89) | 1945 (6.8) | 480 (6.7) | 1.01 (0.99-1.05) | 0.91 (0.85-0.95) |
| Airplane travel | 1066 (2.4) | 243 (2.2) | 1.10 (1.05-1.15) | 1.07 (1.00-1.16) | 1176 (3.0) | 243 (2.5) | 1.21 (1.16-1.27) | 1.00 (0.94-1.10) | 2127 (7.4) | 261 (3.6) | 2.12 (2.06-2.20) | 1.53 (1.43-1.63) |
| Boat travel | 114 (0.3) | 19 (0.2) | 1.44 (1.26-1.74) | 1.50 (1.23-1.90) | 135 (0.3) | 25 (0.3) | 1.32 (1.14-1.57) | 1.20 (0.99-1.47) | 427 (1.5) | 51 (0.7) | 2.11 (1.95-2.27) | 1.40 (1.28-1.51) |
| Long-distance bus travel | 569 (1.3) | 125 (1.1) | 1.14 (1.06-1.20) | 1.29 (1.17-1.39) | 612 (1.5) | 140 (1.4) | 1.10 (1.01-1.17) | 1.25 (1.15-1.39) | 965 (3.4) | 161 (2.3) | 1.52 (1.43-1.58) | 1.20 (1.12-1.29) |
| Long-distance train travel | 2064 (4.7) | 318 (2.9) | 1.65 (1.60-1.72) | 1.61 (1.52-1.72) | 1940 (4.9) | 339 (3.4) | 1.45 (1.39-1.51) | 1.38 (1.30-1.50) | 1758 (6.1) | 327 (4.6) | 1.36 (1.32-1.42) | 1.25 (1.16-1.32) |
| Car-pooling |  |  |  |  |  |  |  |  |  |  |  |  |
| Car-pooling with relatives | 8737 (19.8) | 2218 (20.1) | 0.98 (0.97-1.00) | 1.10 (1.08-1.14) | 8044 (20.3) | 2125 (21.4) | 0.93 (0.91-0.95) | 1.09 (1.05-1.12) | 5832 (20.4) | 1482 (20.7) | 0.98 (0.96-1.00) | 1.18 (1.15-1.21) |
| Car-pooling booked through platform | 348 (0.8) | 181 (1.6) | 0.48 (0.44-0.52) | 0.49 (0.45-0.55) | 353 (0.9) | 177 (1.8) | 0.49 (0.45-0.54) | 0.59 (0.53-0.66) | 307 (1.1) | 166 (2.3) | 0.46 (0.42-0.49) | 0.45 (0.40-0.50) |
| Taxi | 1839 (4.2) | 415 (3.8) | 1.11 (1.07-1.15) | 1.14 (1.10-1.20) | 1721 (4.3) | 371 (3.7) | 1.17 (1.11-1.22) | 1.11 (1.05-1.19) | 1901 (6.6) | 365 (5.1) | 1.32 (1.29-1.37) | 1.08 (1.03-1.15) |
| **Leisure activities** |  |  |  |  |  |  |  |  |  |  |  |  |
| Movie theatre | 2627 (6.0) | 1007 (9.1) | 0.63 (0.61-0.65) | 0.66 (0.64-0.68) | 2241 (5.7) | 928 (9.4) | 0.58 (0.56-0.60) | 0.67 (0.65-0.71) | 1269 (4.4) | 647 (9.0) | 0.47 (0.45-0.49) | 0.53 (0.51-0.57) |
| Concert | 1680 (3.8) | 219 (2.0) | 1.96 (1.89-2.03) | 2.09 (1.97-2.20) | 1965 (5.0) | 297 (3.0) | 1.69 (1.62-1.76) | 1.68 (1.61-1.78) | 661 (2.3) | 119 (1.7) | 1.40 (1.30-1.48) | 1.45 (1.34-1.58) |
| Museum | 963 (2.2) | 248 (2.2) | 0.97 (0.93-1.03) | 0.91 (0.86-0.98) | 915 (2.3) | 221 (2.2) | 1.04 (0.98-1.08) | 1.13 (1.04-1.23) | 1147 (4.0) | 267 (3.7) | 1.08 (1.03-1.13) | 0.95 (0.89-1.01) |
| Theatre | 1177 (2.7) | 241 (2.2) | 1.23 (1.16-1.30) | 1.20 (1.13-1.28) | 993 (2.5) | 237 (2.4) | 1.04 (0.99-1.12) | 1.04 (0.96-1.11) | 453 (1.6) | 111 (1.6) | 1.02 (0.93-1.10) | 1.25 (1.11-1.35) |
| Indoor sports practice | 4149 (9.4) | 946 (8.6) | 1.11 (1.08-1.14) | 1.11 (1.07-1.14) | 3507 (8.8) | 880 (8.9) | 1.00 (0.96-1.03) | 1.02 (0.98-1.07) | 1634 (5.7) | 509 (7.1) | 0.79 (0.76-0.83) | 0.86 (0.81-0.90) |
| Swimming pool | 2460 (5.6) | 684 (6.2) | 0.89 (0.87-0.92) | 0.82 (0.79-0.85) | 2261 (5.7) | 612 (6.2) | 0.92 (0.89-0.95) | 0.97 (0.92-1.02) | 2084 (7.3) | 595 (8.3) | 0.86 (0.84-0.90) | 0.97 (0.92-1.02) |
| Indoor martial arts facility | 519 (1.2) | 145 (1.3) | 0.89 (0.83-0.95) | 0.93 (0.86-0.99) | 421 (1.1) | 115 (1.2) | 0.92 (0.84-0.98) | 1.01 (0.91-1.10) | 167 (0.6) | 94 (1.3) | 0.44 (0.39-0.50) | 0.58 (0.51-0.67) |
| Indoor gymnastics facility | 1980 (4.5) | 509 (4.6) | 0.97 (0.94-1.00) | 0.92 (0.87-0.96) | 1622 (4.1) | 412 (4.2) | 0.99 (0.94-1.03) | 0.97 (0.91-1.02) | 442 (1.5) | 218 (3.0) | 0.50 (0.47-0.53) | 0.59 (0.54-0.63) |
| Outdoor sports practice | 10074 (22.8) | 2936 (26.6) | 0.82 (0.80-0.83) | 0.77 (0.75-0.79) | 8997 (22.7) | 2727 (27.5) | 0.77 (0.76-0.79) | 0.72 (0.70-0.74) | 6365 (22.2) | 1974 (27.6) | 0.75 (0.73-0.77) | 0.71 (0.69-0.73) |
| Bar | 5696 (12.9) | 1523 (13.8) | 0.92 (0.90-0.95) | 1.00 (0.97-1.03) | 5493 (13.9) | 1736 (17.5) | 0.76 (0.74-0.77) | 0.84 (0.82-0.87) | 4902 (17.1) | 1370 (19.2) | 0.87 (0.86-0.89) | 0.89 (0.86-0.92) |
| Restaurant | 13790 (31.2) | 3615 (32.8) | 0.93 (0.92-0.95) | 0.95 (0.93-0.97) | 13777 (34.7) | 3736 (37.7) | 0.88 (0.86-0.90) | 0.93 (0.91-0.95) | 11357 (39.7) | 3075 (43.0) | 0.87 (0.86-0.89) | 0.85 (0.83-0.88) |
| Night-club | 1032 (2.3) | 195 (1.8) | 1.33 (1.26-1.40) | 1.72 (1.59-1.85) | 958 (2.4) | 249 (2.5) | 0.96 (0.91-1.01) | 1.45 (1.34-1.57) | 724 (2.5) | 167 (2.3) | 1.09 (1.02-1.14) | 1.54 (1.41-1.66) |
| Private party | 4739 (10.7) | 1457 (13.2) | 0.79 (0.76-0.81) | 0.87 (0.84-0.90) | 3467 (8.7) | 1343 (13.5) | 0.61 (0.60-0.63) | 0.75 (0.72-0.78) | 2183 (7.6) | 873 (12.2) | 0.60 (0.58-0.62) | 0.65 (0.62-0.68) |

Legend: Adjusted odds ratios estimated in models adjusted for all variables shown in the table, as well as week of exposure, health status (diabetes, hypertension, respiratory disease, coronary artery disease, immunosuppressive treatment, body-mass index categorized as <18.5 kg/m², [18.5-25[, [25-30[, and ≥30 kg/m²), COVID-19 vaccine status (categorized as number of doses and time since last dose as <90 days, 90-179 days, ≥180 days, with a specific category for participants with missing data for the date of the last dose), past SARS-CoV-2 infection (categorized as 61-180 days prior or over 180 days prior), smoking status, level of education, and professional category (of the person in the household with the highest income for the first two periods, of the participant for the following periods). Empty cells reflect changes of the questionnaire or periods when specific settings were closed.

**Table S4: Exposure prevalence, odds ratios of SARS-CoV-2 infection in univariable and multivariable estimates and *p*­-value for interaction term for dining and partying-related exposures in a case-control study conducted in mainland France (2020-2022) – Results for the first three periods of the study (October 1, 2020 to June 13, 2021)**

| Period (onset date) | | 1: 10/01/2020 | | | | 2: 12/04/2020 | | | | 3: 04/09/2021 | | | |
| --- | --- | --- | --- | --- | --- | --- | --- | --- | --- | --- | --- | --- | --- |
|  | | Cases | Controls | OR (95% CI) | Adjusted OR (95% CI) | Cases | Controls | OR (95% CI) | Adjusted OR (95% CI) | Cases | Controls | OR (95% CI) | Adjusted OR (95% CI) |
| Bar or restaurant | Age < 40 | 644 (29.2) | 89 (16.1) | 2.15 (1.96-2.31) | 2.39 (2.10-2.67) |  |  |  |  |  |  |  |  |
|  | Age ≥ 40 | 1258 (24.7) | 197 (15.5) | 1.79 (1.70-1.89) | 1.75 (1.64-1.87) |  |  |  |  |  |  |  |  |
| *p*-value for interaction |  |  |  | 0.2255 | 0.0588 |  |  |  |  |  |  |  |  |
| Party | Age < 40 | 110 (5.0) | 17 (3.1) | 1.68 (1.37-1.90) | 1.33 (0.99-1.70) | 175 (2.4) | 34 (1.9) | 1.29 (1.12-1.48) | 2.17 (1.82-2.57) | 130 (3.3) | 26 (2.7) | 1.25 (1.10-1.44) | 1.98 (1.67-2.51) |
|  | Age ≥ 40 | 43 (0.8) | 10 (0.8) | 1.08 (0.75-1.43) | 0.73 (0.50-1.03) | 21 (0.2) | 16 (0.5) | 0.33 (0.2-0.45) | 0.57 (0.33-0.83) | 7 (0.1) | 4 (0.3) | 0.44 (0.25-0.62) | 0.74 (0.39-1.14) |
| *p*-value for interaction |  |  |  | 0.3543 | 0.2013 |  |  | 0.0003 | 0.0016 |  |  | 0.098 | 0.2178 |

Legend: Adjusted odds ratios estimated in models similar to those displayed in supplementary tables 1-3. Empty cells reflect changes of the questionnaire or periods when specific settings were closed.

**Table S5: Exposure prevalence, odds ratios of SARS-CoV-2 infection in univariable and multivariable estimates and *p*­-value for interaction term for dining and partying-related exposures in a case-control study conducted in mainland France (2020-2022) – Results for the fourth, fifth, and sixth periods of the study (June 14 to December 19, 2021)**

| Period (onset date) | | 4: 06/14/2021 | | | | 5: 08/14/2021 | | | | 6: 10/02/2021 | | | |
| --- | --- | --- | --- | --- | --- | --- | --- | --- | --- | --- | --- | --- | --- |
|  | | Cases | Controls | OR (95% CI) | Adjusted OR (95% CI) | Cases | Controls | OR (95% CI) | Adjusted OR (95% CI) | Cases | Controls | OR (95% CI) | Adjusted OR (95% CI) |
| Bar | Age < 40 | 2446 (45.4) | 367 (27.3) | 2.22 (2.12-2.32) | 2.17 (1.99-2.36) | 496 (24.9) | 129 (25.9) | 0.95 (0.88-1.01) | 1.23 (1.07-1.43) | 875 (27.8) | 168 (21.3) | 1.42 (1.33-1.50) | 1.60 (1.42-1.77) |
|  | Age ≥ 40 | 1034 (17.6) | 283 (19.3) | 0.89 (0.86-0.93) | 1.04 (0.96-1.11) | 411 (14.5) | 130 (18.4) | 0.76 (0.69-0.80) | 1.35 (1.16-1.46) | 1056 (13.0) | 311 (15.4) | 0.83 (0.78-0.87) | 0.85 (0.79-0.91) |
| *p*-value for interaction |  |  |  | < 0.0001 | < 0.0001 |  |  | 0.1549 | 0.6733 |  |  | < 0.0001 | < 0.0001 |
| Restaurant | Age < 40 | 2980 (55.3) | 733 (54.5) | 1.04 (0.99-1.08) | 0.87 (0.8-0.92) | 854 (42.9) | 248 (49.8) | 0.76 (0.70-0.83) | 1.07 (0.96-1.20) | 1374 (43.6) | 371 (47.1) | 0.87 (0.82-0.92) | 0.76 (0.70-0.83) |
|  | Age ≥ 40 | 2331 (39.6) | 675 (45.9) | 0.77 (0.74-0.81) | 1.03 (0.98-1.09) | 976 (34.5) | 332 (47) | 0.6 (0.57-0.63) | 0.84 (0.77-0.91) | 3090 (38.2) | 817 (40.4) | 0.91 (0.88-0.95) | 0.93 (0.88-0.99) |
| *p*-value for interaction |  |  |  | 0.0006 | 0.0748 |  |  | 0.0763 | 0.1278 |  |  | 0.5744 | 0.0706 |
| Party | Age < 40 | 1462 (27.2) | 135 (10.0) | 3.34 (3.19-3.48) | 3.24 (3.01-3.52) |  |  |  |  |  |  |  |  |
|  | Age ≥ 40 | 426 (7.2) | 81 (5.5) | 1.34 (1.23-1.42) | 1.52 (1.33-1.64) |  |  |  |  |  |  |  |  |
| *p*-value for interaction |  |  |  | < 0.0001 | < 0.0001 |  |  |  |  |  |  |  |  |
| Night-club | Age < 40 |  |  |  |  | 229 (11.5) | 36 (7.2) | 1.68 (1.50-1.85) | 2.13 (1.77-2.49) | 332 (10.5) | 45 (5.7) | 1.95 (1.76-2.14) | 2.36 (2.06-2.74) |
|  | Age ≥ 40 |  |  |  |  | 73 (2.6) | 8 (1.1) | 2.32 (1.89-2.64) | 2.91 (2.25-3.58) | 219 (2.7) | 27 (1.3) | 2.06 (1.84-2.31) | 3.08 (2.62-3.67) |
| *p*-value for interaction |  |  |  |  |  |  |  | 0.4308 | 0.5134 |  |  | 0.8123 | 0.3932 |
| Private party | Age < 40 |  |  |  |  | 270 (13.6) | 107 (21.5) | 0.57 (0.52-0.64) | 0.69 (0.6-0.82) | 711 (22.6) | 180 (22.8) | 0.99 (0.91-1.05) | 0.91 (0.81-1.03) |
|  | Age ≥ 40 |  |  |  |  | 157 (5.5) | 83 (11.7) | 0.44 (0.39-0.49) | 0.58 (0.5-0.66) | 947 (11.7) | 302 (14.9) | 0.75 (0.70-0.80) | 0.75 (0.68-0.81) |
| *p*-value for interaction |  |  |  |  |  |  |  | 0.1593 | 0.4451 |  |  | 0.0257 | 0.1543 |

Legend: Adjusted odds ratios estimated in models similar to those displayed in supplementary tables 1-3. Empty cells reflect changes of the questionnaire or periods when specific settings were closed.

**Table S6: Exposure prevalence, odds ratios of SARS-CoV-2 infection in univariable and multivariable estimates and *p*­-value for interaction term for dining and partying-related exposures in a case-control study conducted in mainland France (2020-2022) – Results for the last three periods of the study (December 20, 2021 to October 2, 2022)**

| Period (onset date) | | 7: 12/20/2021 | | | | 8: 03/18/2022 | | | | 9: 05/20/2022 | | | |
| --- | --- | --- | --- | --- | --- | --- | --- | --- | --- | --- | --- | --- | --- |
|  | | Cases | Controls | OR (95% CI) | Adjusted OR (95% CI) | Cases | Controls | OR (95% CI) | Adjusted OR (95% CI) | Cases | Controls | OR (95% CI) | Adjusted OR (95% CI) |
| Bar | Age < 40 | 1993 (17.5) | 463 (16.2) | 1.09 (1.05-1.14) | 1.31 (1.21-1.39) | 1933 (18.8) | 538 (20.9) | 0.88 (0.84-0.91) | 0.95 (0.90-1.02) | 1533 (20.1) | 416 (21.8) | 0.90 (0.87-0.94) | 0.95 (0.89-1.03) |
|  | Age ≥ 40 | 3703 (11.3) | 1060 (13.0) | 0.86 (0.83-0.88) | 0.90 (0.87-0.93) | 3560 (12.1) | 1198 (16.3) | 0.71 (0.68-0.73) | 0.81 (0.78-0.83) | 3369 (16.1) | 954 (18.2) | 0.86 (0.84-0.88) | 0.87 (0.84-0.91) |
| *p*-value for interaction |  |  |  | 0.0003 | < 0.0001 |  |  | 0.0014 | 0.0439 |  |  | 0.5259 | 0.3787 |
| Restaurant | Age < 40 | 3637 (31.9) | 1006 (35.2) | 0.86 (0.83-0.89) | 0.82 (0.78-0.86) | 3738 (36.4) | 1046 (40.7) | 0.83 (0.80-0.86) | 0.81 (0.78-0.86) | 3186 (41.7) | 928 (48.6) | 0.76 (0.73-0.78) | 0.72 (0.68-0.75) |
|  | Age ≥ 40 | 10152 (31.0) | 2609 (31.9) | 0.96 (0.94-0.98) | 1.00 (0.97-1.02) | 10039 (34.2) | 2690 (36.6) | 0.90 (0.88-0.92) | 0.97 (0.94-1.00) | 8171 (39.0) | 2147 (40.9) | 0.92 (0.90-0.94) | 0.90 (0.88-0.93) |
| *p*-value for interaction |  |  |  | 0.0283 | 0.0013 |  |  | 0.1613 | 0.0043 |  |  | 0.0012 | 0.001 |
| Night-club | Age < 40 | 446 (3.9) | 111 (3.9) | 1.00 (0.92-1.10) | 1.23 (1.12-1.41) | 538 (5.2) | 123 (4.8) | 1.10 (1.03-1.19) | 1.76 (1.55-1.95) | 411 (5.4) | 96 (5.0) | 1.07 (0.97-1.16) | 1.46 (1.29-1.63) |
|  | Age ≥ 40 | 586 (1.8) | 84 (1.0) | 1.76 (1.63-1.90) | 2.17 (1.99-2.36) | 421 (1.4) | 126 (1.7) | 0.84 (0.74-0.92) | 1.15 (1.01-1.27) | 313 (1.5) | 71 (1.4) | 1.10 (0.99-1.19) | 1.67 (1.42-1.88) |
| *p*-value for interaction |  |  |  | 0.0005 | 0.002 |  |  | 0.0607 | 0.0147 |  |  | 0.7948 | 0.5111 |
| Private party | Age < 40 | 1695 (14.9) | 492 (17.2) | 0.84 (0.79-0.88) | 0.96 (0.89-1.01) | 1188 (11.6) | 432 (16.8) | 0.65 (0.60-0.68) | 0.76 (0.71-0.82) | 804 (10.5) | 312 (16.3) | 0.6 (0.56-0.64) | 0.69 (0.62-0.74) |
|  | Age ≥ 40 | 3044 (9.3) | 965 (11.8) | 0.77 (0.74-0.79) | 0.83 (0.80-0.87) | 2280 (7.8) | 911 (12.4) | 0.59 (0.57-0.62) | 0.74 (0.71-0.78) | 1379 (6.6) | 561 (10.7) | 0.59 (0.56-0.62) | 0.64 (0.60-0.67) |
| *p*-value for interaction |  |  |  | 0.1908 | 0.0756 |  |  | 0.2608 | 0.6694 |  |  | 0.6956 | 0.4368 |

Legend: Adjusted odds ratios estimated in models similar to those displayed in supplementary tables 1-3. Empty cells reflect changes of the questionnaire or periods when specific settings were closed.
